# Development and preclinical evaluation of selective vinyl sulfone-based probes for PET imaging of tumor-associated cathepsins

**DOI:** 10.1186/s41181-026-00493-5

**Published:** 2026-08-31

**Authors:** Oliviero Cini, Alan Miranda, Daan Willocx, Angeliki Karakasidi, Anke de Groot, Thales do Valle Moreira, Ana P. Leandro, Maria M. M. Santos, Rui Moreira, Filipe Elvas

**Affiliations:** 1 Research Institute for Medicines (iMed.ULisboa), Faculty of Pharmacy, Universidade de Lisboa, Av. Prof. Gama Pinto, Lisboa, 1649-003 Portugal; 2 Molecular Imaging and Radiology, University of Antwerp, Universiteitsplein 1, 2610 Wilrijk, Antwerpen, 2610 Belgium; 3 Institute of Pharmacy and Biochemistry, University of Mainz, Staudingerweg 5, 55128 Mainz, Germany

**Keywords:** Cysteine cathepsins, Cathepsin L, Vinyl sulfones, Activity-based probes, Radiotracer, PET imaging

## Abstract

**Background:**

Cysteine cathepsin L (CatL) is emerging as a key biomarker of cancer progression, making it an attractive candidate for non-invasive molecular imaging. To monitor enzymatic activity directly, rather than merely protein expression, activity-based probes (ABPs) enable selective targeting of enzymes through covalent binding at their active sites. Despite vinyl sulfones are well-established irreversible inhibitors of cathepsins, their application as positron emission tomography (PET) ABPs remains unexplored. In this study we describe the design, synthesis, and evaluation of a novel class of ^68^Ga-labeled vinyl sulfone-based ABPs targeting CatL.

**Results:**

A series of ABPs were synthesized based on the established vinyl sulfone scaffold K11777 (K777). Structure-activity relationship (SAR) analysis revealed that retaining a homophenylalanine (hPhe) residue at the P1 position resulted in greater CatL potency compared to its corresponding leucine (Leu) analogue. Evaluation of different linkers at the P3 position further revealed that polyethylene glycol (PEG) linkers preserved high CatL selectivity, whereas a piperazine linker increased off-target binding to cathepsin B (CatB). Based on these findings, two representative radiotracers were selected for further biological evaluation: the PEG2-linked [^68^Ga]CREANT-101 and the piperazine-linked [^68^Ga]CREANT-102. In vivo pharmacokinetic studies in healthy mice demonstrated that [^68^Ga]CREANT-102 exhibited prolonged blood circulation and high non-specific tissue uptake, whereas [^68^Ga]CREANT-101 displayed rapid systemic clearance and low background signal, supporting its selection for tumor imaging. In both U-87 MG and HT-29 xenograft models, [^68^Ga]CREANT-101 showed modest absolute tumor uptake in vivo. However, subsequent ex vivo analysis of tumor homogenates confirmed successful and specific engagement of active cathepsins within the target tissue.

**Conclusions:**

A novel class of ^68^Ga-labeled vinyl sulfone ABPs targeting CatL was successfully developed and preclinically evaluated. The lead tracer, [^68^Ga]CREANT-101, demonstrated high selectivity and a favorable background profile, but showed limited tumor accumulation in vivo. Ex vivo findings indicated that this limited tumor uptake was not attributable to insufficient target affinity, but rather to suboptimal cellular internalization and restricted tumor bioavailability, likely resulting from rapid systemic clearance and in vivo metabolic instability. Therefore, future optimization of this tracer class should focus on improving these pharmacokinetic properties to enhance in vivo tumor targeting.

**Supplementary Information:**

The online version contains supplementary material available at https://doi.org/10.1186/s41181-026-00493-5.

## Background

Cancer remains a major global health challenge, underscoring the urgent need for advanced diagnostic tools capable of early detection and of supporting of precision oncology (Bray et al. 2024; Gandhi et al. 2025; Velikyan 2015, 2026). PET is a highly sensitive, non-invasive molecular imaging modality that allows real-time assessment and quantification of biological activity at both molecular and cellular levels. (Crișan et al. 2022; Gandhi et al. 2025). Gallium-68 (^68^Ga) is a positron (β^+^) emitter that has become established as a valuable radiometal for PET applications due to its favorable physical half-life (67.7 min), widespread availability via generator systems, and compatibility with robust bifunctional chelators. These properties make it well suited for labeling low-molecular-weight radiopharmaceuticals for clinical use (Banerjee and Pomper 2013; Baum and Rösch 2013; Davey and Paterson 2022; Tanwar and Pandey 2025). In the search for reliable oncological biomarkers, cysteine cathepsins have gained significant attention. Once regarded primarily as lysosomal degradative enzymes, these proteases are now recognized as key contributors to tumor progression, angiogenesis, evasion of apoptosis, and metastasis (Brömme and Wilson 2011; Lankelma et al. 2010; Mohamed and Sloane 2006; Olson and Joyce 2015; Vidak et al. 2019; Yadati et al. 2020). During malignant transformation, cathepsins are often upregulated and secreted into the acidic tumor microenvironment (TME), where they are involved in processes such as extracellular matrix (ECM) degradation and tumor invasion (Mohamed and Sloane 2006; Olson and Joyce 2015). Within this enzyme family, endopeptidase cathepsin L (CatL) is involved in multiple stages of cancer progression, contributing to the degradation of ECM and adhesion molecules, thereby promoting invasion and angiogenesis, and is expressed by different tumor cell types (Mohamed and Sloane 2006; Olson and Joyce 2015; Vidak et al. 2019). CatL activity is also strongly associated with advanced tumor grade and unfavorable prognosis in a variety of solid malignancies, underscoring its relevance as a molecular target for non-invasive imaging approaches (Berdowska 2004; Biasizzo et al. 2022; Garland et al. 2016; Kramer et al. 2017; Löser and Pietzsch 2015; Olson and Joyce 2015; Rot et al. 2024; Schleyer and Cui 2021). Consequently, targeting CatL with PET radiotracers offers a promising strategy for early cancer detection and the monitoring of tumor progression (Biasizzo et al. 2022; Hughes et al. 2016; Rot et al. 2024).

To directly monitor enzyme activity, rather than simple expression levels, ABPs have been developed. ABPs are small molecules used in chemical proteomics and cell imaging that covalently target catalytic amino acid residues via an electrophilic group and enable detection and enrichment through an attached reporter tag (Ahmadi and Emami 2026; Dana and Pathak 2020; Garland et al. 2016; Nguyen et al. 2025; Rot et al. 2024). Depending on their design, such probes can be adapted to different imaging modalities, although only a limited number of PET tracers targeting cathepsins have been reported. Among the most notable examples are dual-modality ABPs, incorporating both a radionuclide and a fluorophore, such as the pan-cathepsin tracers [^64^Cu]GB170 (Ren et al. 2011) and [^68^Ga]BMV101 (Withana et al. 2016), as well as the CatB-selective [^68^Ga]/[^90^Y]BMX2 (Zhou et al. 2023); activatable substrate-based tracers, reported for CatB (Li et al. 2024); and radiolabeled small-molecule inhibitors, developed for CatK (Bennacef et al. 2021; Rodnick et al. 2014) and, more recently, CatS (Yang et al. 2026).

**Fig. 1:**
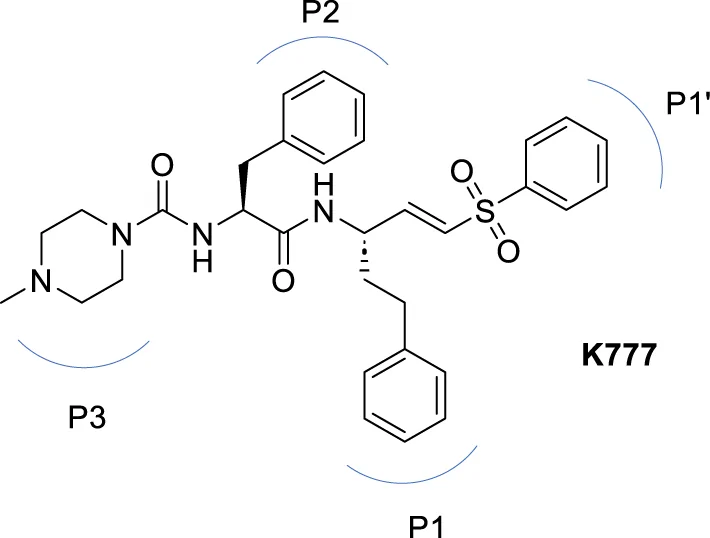
Chemical structure of the cysteine protease inhibitor K777, with the enzyme subsites occupied by each fragment indicated: the N-methylpiperazine (P3), the Phe (P2) and hPhe (P1) residues of the dipeptide recognition element, and the phenyl vinyl sulfone warhead (P1′)

Among available warheads, vinyl sulfones are particularly attractive for in vivo applications. As Michael acceptors, they react covalently with catalytic cysteine residues, while exhibiting enhanced metabolic stability compared to other electrophilic warheads, such as acrylamides and epoxides (Ahmadi and Emami 2026; Dunny et al. 2013; Jaishankar et al. 2008; Palmer et al. 1995; Powers et al. 2002). A prototypical example is K777, a well-validated dipeptide vinyl sulfone-based inhibitor with high potency toward human CatL and CatB (Fig. 1) (Jung et al. 2022; Latorre et al. 2016; Mellott et al. 2021).

The K777 scaffold incorporates a dipeptide fragment (hPhe – Phe) that mimics substrate recognition at the non-primed P1 and P2 positions, complemented by a phenyl vinyl sulfone (P1’) and an N-methyl piperazine moiety (P3) (Wang et al. 2023). Specifically, the solvent-exposed P3 position represents a versatile anchoring site for the introduction of linkers and reporter groups, enabling the conversion of the inhibitor into a PET probe with minimal disruption of enzyme binding. At the same time, the synthetic versatility of the scaffold allows fine-tuning of its affinity through the incorporation of different aminoacidic residues. While vinyl sulfones are a well-validated inhibitor class for cathepsins, their potential as PET ABPs remains unexplored. Moreover, although targeting cathepsins holds significant potential, achieving high in vivo selectivity and favorable pharmacokinetic profiles remains a significant challenge (Hughes et al. 2016; Schleyer and Cui 2021). Consequently, expanding the chemical space of cathepsin-targeted radiotracers is essential. In this work, we present the design, synthesis, and evaluation of a novel class of ^68^Ga-labeled PET ABPs targeting CatL. This proof-of-concept study aims to explore the feasibility of vinyl sulfone-based scaffolds as PET ABPs and assess their pharmacokinetic profile.

Using the validated K777 vinyl sulfone scaffold, we introduced modifications at two distinct sites. To isolate linker effects, a series of distinct spacer linkers was introduced at the P3 position while retaining the core scaffold. Additionally, inspired by recent reports showing that potent CatL inhibitors often feature a leucine in P1 position across different warheads (Dana et al. 2014; Fernández-de-la-Pradilla et al. 2023), a specific Leu-containing analogue at P1 was synthesized. This approach enabled us to assess how linker flexibility and properties and P1 residue variations independently impact the balance between in vitro enzymatic affinity and in vivo pharmacokinetic performance. To enable radiolabeling, the linkers were conjugated with NODA-GA, a macrocyclic chelator selected over alternative chelators like DOTA and NOTA due to its ability to form highly stable and kinetically robust complexes with ^68^Ga (Ashhar et al. 2020; Fani et al. 2011; Patra et al. 2019; Poty et al. 2016; Satpati et al. 2018; Sun et al. 2016). Through this rational design, we aim to develop selective and stable PET radiotracers capable of non-invasively mapping tumor-associated CatL activity, expanding the diagnostic toolkit available for precision oncology to support accurate disease detection, staging, and monitoring.

## Methods

### General

The reagents and solvents used were purchased from commercial suppliers and used without further purification. Reactions under N_2_ atmosphere were performed in oven-dried glassware and anhydrous solvents. Nuclear Magnetic Resonance (NMR) spectra were recorded on Bruker Avance 300 (300 MHz for ^1^H, 75 MHz for ^13^C), Bruker Avance 400 (400 MHz for ^1^H, 101 MHz for ^13^C) or Bruker Avance III 500 (500 MHz for ^1^H, 126 MHz for ^13^C) spectrometer and analyzed with MestReNova software. Flash column chromatography was performed using silica gel 60 (230–400 mesh ASTM, Merk). Preparative thin-layer chromatography (PTLC) was performed on 0.5 and 1 mm-thick silica gel 60 G F_254_ (Merck) layers on glass plates. Purifications of probe precursors were carried out using a CombiFlash system (Teledyne ISCO) operating a two different wavelength (220–254 nm) on a RediSep Gold C18Aq column (Teledyne ISCO). Analytical high-performance liquid chromatography (HPLC) of the non-radioactive ^nat^Ga complexes was performed on an Elite LaChrom (VWR Hitachi) system equipped with an L-2130 pump and an L-2400 UV detector set at 230 nm. Separation was achieved on a Lichrospher 100 RP-18 (5 μm) column at a flow rate of 1.0 mL/min, using an aqueous solution of TFA (0.1% v: v) (A) and an ACN solution of TFA (0.1%, v: v) (B) with the following gradient: 0–5 min, 90% A; 5–10 min, 90%–40% A; 10–20 min, 40% A; 20–25 min, 40%–10% A; 25–30 min, 10% A. Low resolution mass spectra (LRMS) were recorded on a MicroMass Quattromicro API triple quadrupole (Waters). The ionization of the compounds was performed using an electrospray ionization (ESI) source in both positive and negative modes (ESI + and ESI-). High resolution ESI mass spectra (HRMS) on positive mode were recorded using Exactive PLUS Hybrid Quadrupole-Orbitrap (Thermo Fisher).

For ^68^Ga radiolabeling radio-HPLC analyses were carried out using an analytical radio-HPLC system consisting of a Nexera-i system (Shimadzu), coupled to a single-quadrupole LCMS-2020 mass spectrometer equipped with an ESI interface (Shimadzu) and a GABI Nova radio-flow monitor (Raytest). Chromatographic separation was performed by using a Phenomenex Kinetex C18 (2.6 μm, 100 × 3 mm, 2.6 μm) column (Phenomenex). A gradient of aqueous solution of formic acid (0.1%, v: v) (A) and ACN solution of formic acid (0.1%, v: v) (B) was applied at a mobile phase flow rate of 0.5 mL/min: 0–15 min, 10%–90% B. The oven temperature was set at 40 °C. MS conditions were as follows: full scan acquisition, both ESI + and ESI − in the m/z range 100–1200. For in vitro mouse plasma stability and in vivo metabolic stability, analyses were carried out using a Shimadzu LC20 HPLC system (Shimadzu) equipped with a SPD-20AV UV–VIS detector (Shimadzu), coupled in series with a NaI scintillation detector 2 × 2″ pinhole (Raytest) and a GABI Star isotope measuring system (Raytest).

### Chemistry

The synthesis and characterization of compounds **1**–**14** can be found in the Supporting Information. Weinreb amides **1**–**2**, aldehydes **3**–**4**, sulfone **5**, vinyl sulfones **6**–**7**, and dipeptidyl vinyl sulfones **8–9** were synthesized according to the previously published synthetic protocols (Capela et al. 2009).

Compounds **1**–**6** and **8** are known compounds, and their NMR data were in good agreement with those reported in the literature: compounds **1**, **3**, **6** and **8** (Roush et al. 1998), compound **5** (Fuchs et al. 2023), and compounds **2** and **4** (Fernández-de-la-Pradilla et al. 2023).

### General procedure for the synthesis of probe precursors

A solution of Boc–linker–dipeptidyl vinyl sulfone (0.03 mmol, 1 mol. equiv.) in dichloromethane (1 mL) and trifluoroacetic acid (1 mL) was stirred at room temperature for 3 h. The solvent was then removed under reduced pressure, and the residue was kept under high vacuum until further use.

The resulting deprotected linker–dipeptidyl vinyl sulfone was dissolved in dry dimethylformamide (2 mL), and N, N-diisopropylethylamine (0.29 mmol, 9.5 mol. equiv.) was added dropwise. Subsequently, NODA-GA NHS ester (0.05 mmol, 1.5 mol. equiv.) was added, and the reaction mixture was stirred for 16 h at room temperature under a nitrogen atmosphere. After removal of the dimethylformamide under reduced pressure, the crude product was purified by automated flash chromatography. The fractions containing the compound were combined and concentrated under reduced pressure to afford the pure compound.

**2**,**2’-(7-((4S**,**7S)-7-benzyl-21-carboxy-2-methyl-6**,**9**,**18-trioxo-4-((E)-2-(phenylsulfonyl)vinyl)-11**,**14-dioxa-5**,**8**,**17-triazahenicosan-21-yl)-1**,**4**,**7-triazonane-1**,**4-diyl)diacetic acid − 15**.

White solid (7 mg, 35% yield). ^1^H NMR (500 MHz, MeOD) *δ* = 7.89–7.81 (m, 2 H), 7.74–7.67 (m, 1H), 7.66–7.59 (m, 2 H), 7.25–7.14 (m, 5 H), 6.79 (d, *J* = 15.0, 4.9 Hz, 1H), 6.04 (dd, *J* = 15.1, 1.8 Hz, 1H), 4.68–4.57 (m, 2 H), 4.01–3.90 (m, 2 H), 3.88–3.73 (m, 4 H), 3.66–3.51 (m, 7 H), 3.50–2.77 (m, 16 H), 2.48–2.30 (m, 2 H), 2.17–2.07 (m, 1H), 2.05–1.92 (m, 1H), 1.66–1.57 (m, 1H), 1.48–1.34 (m, 2 H), 0.89 (d, *J* = 6.7 Hz, 3 H), 0.87 (d, *J* = 6.5 Hz, 3 H) ppm. ^13^C NMR (126 MHz, MeOD) *δ* = 175.3, 175.1, 172.7, 172.3, 148.1, 141.8, 137.7, 134.8, 131.0, 130.6, 130.5, 129.7, 128.7, 128.3, 72.0, 71.1, 71.0, 70.8, 64.8, 56.4, 55.6, 52.2, 50.4, 49.8, 43.3, 40.3, 39.3, 33.6, 26.6, 25.8, 23.3, 21.9 ppm. HRMS *m/z* (ESI^+^): 903.4176 [M + H]^+^. Calculated for C_43_H_62_N_6_O_13_S 903.4169.

**2**,**2’-(7-((3S**,**6S**,**E)-6-benzyl-17-carboxy-5**,**8**,**14-trioxo-3-phenethyl-1-(phenylsulfonyl)-10-oxa-4**,**7**,**13-triazaheptadec-1-en-17-yl)-1**,**4**,**7-triazonane-1**,**4-diyl)diacetic acid – 16**.

White solid (8 mg, 33% yield). ^1^H NMR (500 MHz, MeOD) *δ* = 7.88–7.83 (m, 2 H), 7.74–7.67 (m, 1H), 7.66–7.59 (m, 2 H), 7.27–7.11 (m, 10 H), 6.78 (dd, *J* = 15.1, 4.8 Hz, 1H), 6.05 (d, *J* = 15.0 Hz, 1H), 4.67–4.60 (m, 1H), 4.53–4.46 (m, 1H), 4.02–3.92 (m, 2 H), 3.89–3.63 (m, 4 H), 3.57–3.50 (m, 3 H), 3.42–3.37 (m, 2 H), 3.30–2.94 (m, 14 H), 2.69–2.61 (m, 1H), 2.61–2.52 (m, 1H), 2.48–2.41 (m, 2 H), 2.21–2.09 (m, 1H), 2.04–1.96 (m, 1H), 1.95–1.87 (m, 1H), 1.84–1.74 (m, 1H) ppm. ^13^C NMR (126 MHz, MeOD) *δ* = 175.4, 175.2, 173.0, 172.2, 147.5, 142.2, 141.8, 137.9, 134.8, 131.5, 130.6, 130.4, 129.7, 129.6, 129.5, 128.7, 128.2, 127.2, 71.3, 71.0, 65.0, 56.9, 56.0, 52.2, 50.7, 40.2, 39.0, 36.3, 33.5, 33.0, 26.5 ppm. HRMS *m/z* (ESI^+^): 907.3915 [M + H]^+^. Calculated for C_45_H_58_N_6_O_12_S 907.3906.

**2**,**2’-(7-((3S**,**6S**,**E)-6-benzyl-20-carboxy-5**,**8**,**17-trioxo-3-phenethyl-1-(phenylsulfonyl)-10**,**13-dioxa-4**,**7**,**16-triazaicos-1-en-20-yl)-1**,**4**,**7-triazonane-1**,**4-diyl)diacetic acid – 17**.

White solid (10 mg, 30% yield). ^1^H NMR (500 MHz, MeOD) *δ* = 7.89–7.81 (m, 2 H), 7.74–7.67 (m, 1H), 7.66–7.58 (m, 2 H), 7.28–7.08 (m, 10 H), 6.79 (d, *J* = 15.4 Hz, 1H), 6.03 (d, *J* = 15.2 Hz, 1H), 4.72–4.63 (m, 1H, ), 4.55–4.46 (m, 1H), 4.05–3.91 (m, 2 H), 3.83–3.67 (m, 4 H), 3.66–3.52 (m, 7 H), 3.42–2.81 (m, 16 H), 2.69–2.61 (m, 1H), 2.60–2.52 (m, 1H), 2.44–2.37 (m, 2 H), 2.22–2.01 (m, 1H), 1.99–1.87 (m, 2 H), 1.84–1.72 (m, 1H) ppm. ^13^C NMR (126 MHz, MeOD) *δ* = 175.2, 172.4, 147.5, 142.2, 141.8, 137.8, 134.8, 131.5, 130.6, 130.5, 129.7, 129.6, 129.5, 128.7, 128.3, 127.2, 72.1, 71.1, 71.0, 70.8, 65.1, 56.9, 55.8, 52.3, 50.7, 40.3, 39.3, 36.3, 33.6, 33.0, 26.5 ppm. HRMS *m/z* (ESI^+^): 951.4187 [M + H]^+^. Calculated for C_47_H_62_N_6_O_13_S 951.4169.

**2**,**2’-(7-((3S**,**6S**,**E)-6-benzyl-23-carboxy-5**,**8**,**20-trioxo-3-phenethyl-1-(phenylsulfonyl)-10**,**13**,**16-trioxa-4**,**7**,**19-triazatricos-1-en-23-yl)-1**,**4**,**7-triazonane-1**,**4-diyl)diacetic acid – 18**.

White solid (5 mg, 28% yield). ^1^H NMR (500 MHz, MeOD): δ 7.88–7.82 (m, 2 H), 7.73–7.68 (m, 1H), 7.65–7.60 (m, 2 H), 7.27–7.10 (m, 10 H), 6.79 (dd, *J* = 15.1, 4.9 Hz, 1H), 6.04 (dd, *J* = 15.0, 1.8 Hz, 1H), 4.68–4.62 (m, 1H), 4.53–4.48 (m, 1H), 4.06–3.94 (m, 2 H), 3.83–3.70 (m, 4 H), 3.69–3.58 (m, 9 H), 3.57–2.77 (m, 18 H), 2.68–2.60 (m, 1H), 2.60–2.52 (m, 1H), 2.44–2.37 (m, 2 H), 2.16–2.04 (m, 1H), 2.01–1.87 (m, 2 H), 1.84–1.72 (m, 1H) ppm. ^13^C NMR (126 MHz, MeOD) *δ* = 172.9, 172.5, 147.5, 142.2, 141.8, 137.8, 134.8, 131.5, 130.6, 130.5, 129.7, 129.6, 129.5, 128.7, 128.3, 127.2, 71.9, 71.6, 71.4, 71.2, 71.1, 70.6, 65.2, 56.8, 55.9, 52.3, 50.7, 40.4, 39.1, 36.3, 33.5, 33.0, 26.4 ppm. HRMS *m/z* (ESI^+^): 995.4447 [M + H]^+^. Calculated for C_49_H_66_N_6_O_14_S 995.4431.

**2**,**2’-(7-(1-carboxy-4-oxo-4-(4-(((S)-1-oxo-3-phenyl-1-(((S**,**E)-5-phenyl-1-(phenylsulfonyl)pent-1-en-3-yl)amino)propan-2-yl)carbamoyl)piperazin-1-yl)butyl)-1**,**4**,**7-triazonane-1**,**4-diyl)diacetic acid − 19**.

White solid (11 mg, 50% yield). ^1^H NMR (500 MHz, MeOD) *δ* = 7.88–7.82 (m, 2 H), 7.73–7.67 (m, 1H), 7.65–7.59 (m, 2 H), 7.27–7.10 (m, 10 H), 6.79 (dd, *J* = 15.1, 4.5 Hz, 1H), 6.08 (d, *J* = 15.1 Hz, 1H), 4.55–4.41 (m, 2 H), 4.05–2.78 (m, 27 H), 2.74–2.48 (m, 4 H), 2.14–1.97 (m, 2 H), 1.96–1.86 (m, 1H), 1.85–1.74 (m, 1H) ppm. ^13^C NMR (126 MHz, MeOD) *δ* = 175.2, 174.7, 173.5, 173.1, 159.2, 147.6, 142.3, 141.8, 138.5, 134.8, 131.4, 130.6, 130.4, 129.6, 129.6, 129.5, 128.7, 128.1, 127.1, 64.6, 58.1, 56.4, 50.6, 46.2, 44.6, 42.5, 39.1, 36.4, 32.9, 30.9, 26.0 ppm. HRMS *m/z* (ESI^+^): 918.4045 [M + H]^+^. Calculated for C_46_H_59_N_7_O_11_S 918.4066.

### General procedure for the synthesis of non-radioactive complexes

The probe precursor (1 µmol, 1 mol. equiv.) was first dissolved in DMSO to a final concentration of 20 mM and subsequently diluted with 500 µL of 1 M NH_4_OAc buffer at pH 5.5. A 50 mM solution of ^nat^GaCl_3_ (5 µmol, 5 mol. equiv.) was then added, and the mixture was stirred at 25 °C for 15 min. Purification was performed using a Sep-Pak tC2 Plus Light cartridge, washing with 5 mL of water and subsequently eluting the compound with 1 mL of acetonitrile. Chromatograms of the ^nat^Ga complexes **20–24** are provided in the Supporting Information (Supplemental Figures 2–6).

#### Gallium 2,2’-(7-((4S,7S)-7-benzyl-21-carboxylato-2-methyl-6,9,18-trioxo-4-((E)-2-(phenylsulfonyl)vinyl)-11,14-dioxa-5,8,17-triazahenicosan-21-yl)-1,4,7-triazonane-1,4-diyl)diacetate – 20

White solid (0.5 mg, 97% yield). HRMS m/z (ESI+): 969.3187 [M + H]+. Calculated for C_43_H_59_GaN_6_O_13_S: 969.3189.

#### Gallium 2,2’-(7-((3S,6S,E)-6-benzyl-17-carboxylato-5,8,14-trioxo-3-phenethyl-1-(phenylsulfonyl)-10-oxa-4,7,13-triazaheptadec-1-en-17-yl)-1,4,7-triazonane-1,4-diyl)diacetate – 21

White solid (1 mg, 93% yield). HRMS m/z (ESI+): 973.2918 [M+H]+. Calculated for C_45_H_55_GaN_6_O_12_S: 973.2927.

#### Gallium 2,2’-(7-((3S,6S,E)-6-benzyl-20-carboxylato-5,8,17-trioxo-3-phenethyl-1-(phenylsulfonyl)-10,13-dioxa-4,7,16-triazaicos-1-en-20-yl)-1,4,7-triazonane-1,4-diyl)diacetate – 22

White solid (0.7 mg, 96% yield). HRMS m/z (ESI+): 1017.3177 [M+H]+. Calculated for C_47_H_59_GaN_6_O_13_S: 1017.3189.

#### Gallium 2,2''''-(7-((3S,6S,E)-6-benzyl-23-carboxylato-5,8,20-trioxo-3-phenethyl-1-(phenylsulfonyl)-10,13,16-trioxa-4,7,19-triazatricos-1-en-23-yl)-1,4,7-triazonane-1,4-diyl)diacetate - 23

White solid (0.8 mg, 96% yield). HRMS m/z (ESI+): 1061.3457 [M+H]+. Calculated for C_49_H_63_GaN_6_O_14_S: 1061.3452.

#### Gallium 2,2''''-(7-(1-carboxylato-4-oxo-4-(4-(((S)-1-oxo-3-phenyl-1-(((S,E)-5-phenyl-1-(phenylsulfonyl)pent-1-en-3-yl)amino)propan-2-yl)carbamoyl)piperazin-1-yl)butyl)-1,4,7-triazonane-1,4-diyl)diacetate - 24

White solid (0.6 mg, 98% yield). HRMS m/z (ESI+): 984.3073 [M+H]+. Calculated for C_46_H_56_GaN_7_O_11_S: 984.3087.

### Fluorometric enzyme assays

#### Assay procedure

Fluorometric assays were performed on FLUOstar Omega (BMG Labtech) microplate reader by monitoring the increase in fluorescence (excitation: 360 nm, emission: 460 nm) resulting from the release of AMC from the fluorogenic substrate Z-FR-AMC (Bachem) by CatB (Calbiochem) or CatL (Calbiochem). Enzyme stock solutions (CatB: 0.379 mg/mL; CatL: 0.224 mg/mL) were diluted in assay buffer immediately before use. For CatB, the buffer consisted of MES hydrate 100 mM, EDTA 2.5 mM, DTT 2.5 mM, Tween 20 0.001%, pH 6, whereas CatL assays were carried out in MES hydrate 100 mM, EDTA 2.5 mM, DTT 2.5 mM, pH 5.5. Enzymatic assays were conducted at 37 °C in white 96-well flat-bottom plates in a final reaction volume of 200 µL, with final enzyme concentrations of 2.75 nM for CatB and 3.85 nM for CatL. For each specific inhibitor, seven concentrations were prepared by serial dilution and analyzed in triplicate. For each condition, a mixture of 10 µL of inhibitor solution in DMSO or DMSO control together with 5 µL substrate solution in DMSO were transferred into an RT-PCR plate. Subsequently, 185 µL of enzyme solution was dispensed into each well of the assay plate. Reactions were initiated by adding 15 µL of the inhibitor/substrate mixture from the RT-PCR plate to the enzyme-containing wells, and fluorescence acquisition started immediately. Final substrate concentrations were 100 µM for CatB and 6.25 µM for CatL, while the final DMSO concentration was maintained constant at 7.5% (v/v) in all assay wells. Data are reported as the mean of three independent measurements.

#### K_I_ calculation

Progress curves corresponding to time-dependent inhibition at each inhibitor concentration were individually fitted to a general exponential equation (Eq. 1) for inhibitor association to determine the observed rate of inactivation *k*_obs_:

1 $$\:\begin{array}{c}{F}_{t}=\:{F}_{0}+\:{\mathrm{v}}_{\mathrm{s}}t+\:\frac{{\mathrm{v}}_{\mathrm{i}}-\:{\mathrm{v}}_{\mathrm{s}}}{{k}_{\mathrm{o}\mathrm{b}\mathrm{s}}}\:\left[1-\:{e}^{-{k}_{\mathrm{o}\mathrm{b}\mathrm{s}}t}\right]\end{array}$$

The resulting *k*_obs_ values were subsequently plotted against the inhibitor concentration [I] and fitted to determine the maximum inactivation rate constant *k*_inact_ and the apparent inactivation constant K_I_^app^ using the following equation (Eq. 2):

2 $$\:\begin{array}{c}{k}_{\mathrm{o}\mathrm{b}\mathrm{s}}=\:\frac{{k}_{\mathrm{i}\mathrm{n}\mathrm{a}\mathrm{c}\mathrm{t}\:}\left[\mathrm{I}\right]}{{\mathrm{K}}_{\mathrm{I}}^{\:\mathrm{a}\mathrm{p}\mathrm{p}}+\left[\mathrm{I}\right]}\end{array}$$

To account for substrate competition, K_I_^app^ values were corrected using the Cheng-Prusoff equation (Eq. 3) to obtain inactivation constant K_I_ (Cheng and Prusoff):

3 $$\:\begin{array}{c}{\mathrm{K}}_{\mathrm{I}}=\:\frac{{\mathrm{K}}_{\mathrm{I}}^{\mathrm{a}\mathrm{p}\mathrm{p}}}{(\:1+\:\frac{\left[\mathrm{S}\right]}{{\mathrm{K}}_{\mathrm{M}}}\:)}\end{array}$$

Where [S] represents substrate concentration and K_M_ the Michaelis-Menten constant determined under the assay conditions (CatB: [S] = 100 µM, K_M_ = 83 µM; CatL: [S] = 6.25 µM, K_M_ =1.6 µM).

#### K_m_ determination

The assay was performed as described above using different concentrations of Z-FR-AMC. The K_M_ value was calculated as described by Michaelis-Menten equation (Eq. 4):

4 $$\:\begin{array}{c}{v}_{o}=\:\frac{{v}_{max}\:\left[S\right]}{{K}_{M}+\left[S\right]}\end{array}$$

#### Radiosynthesis of [^68^Ga]CREANT-101 and − 102

Radiolabeling was carried out by incubating 50 µg precursor dissolved in 500 µL of metal-free 1 M NH_4_OAc buffer (pH 5.5) with 1 mL of [^68^Ga]Ga^3+^ eluate (800–950 MBq, Galli Ad, IRE ELiT) for 5 min at 80 °C. The reaction was quenched by adding 10 µL of 1% EDTA solution. Purification was performed using a preconditioned Sep-Pak tC2 Plus Light cartridge (Waters). After washing with 5 mL WFI, the product was eluted into a sterile vial using 0.5 mL ethanol and was diluted with 4.5 mL sterile 0.9% NaCl solution. Radiochemical purity (RCP) was evaluated both by radio-HPLC, integrating the area under the curve (AUC), and by radio-iTLC (NH_4_OAc/MeOH 1:1 (v/v) buffer (pH 5.5): [^68^Ga]CREANT-101 and − 102: Rf = 1; 0.1 M sodium citrate buffer (pH 5.5): [^68^Ga]CREANT-101 and − 102: Rf = 0).

#### Partition coefficient determination

The partition coefficient of the radiotracers was determined using the shake-flask method. Approximately 185 kBq of tracer was added to a test tube containing an equal volume (2 mL) of PBS (0.01 M, pH 7.4) and *n*-octanol. Samples were vigorously shaken, vortexed for 2 min, and centrifuged at 3000 rpm for 10 min. After phase separation, aliquots (0.5 mL) of each layer were transferred into separate pre-weighed tubes and measured for radioactivity using an automatic γ-counter (Wizard^2^ 2480, Revvity). Activity values were corrected for density and mass before calculating the octanol/PBS partition coefficient. Log D values were obtained after logarithmic transformation (log_10_), presented as mean ± SD from triplicate measurements for each probe.

#### Activity-based labeling of recombinant cathepsins

Recombinant cathepsins B, L, and S were purchased from R&D Systems. Enzymes were first diluted in 50 µL of cathepsin assay buffer (50 mM sodium acetate, 5 mM MgCl_2_ and 2 mM DTT, pH 5.5) to obtain a final concentration of 20 nM. The enzyme solutions were then incubated with approximately 222 kBq of [^68^Ga]CREANT-101 or -102 for 10 or 60 min at 37 °C, or for 30 min in the case of co-incubation experiments. Where indicated, samples were pre-incubated for 30 min with the pan-cathepsin inhibitor K777 (1 µM). Reactions were quenched by addition of 5× SDS loading buffer (0.25 M Tris-Cl, pH 6.8, 0.05% bromophenol blue, 10% SDS, 50% glycerol, 5% β-mercaptoethanol) followed by heating at 95 °C for 5 min.

Then the samples were loaded onto Mini-PROTEAN TGX gels (Bio-Rad) and separated by SDS-PAGE. Gels were washed and exposed overnight to phosphor imaging plates (Fujifilm), which were subsequently scanned using a FLA7000 phosphor imager (GE Healthcare).

Gel images were processed using ImageJ (v1.54). Images were uniformly adjusted to improve visualization, cropped around the relevant protein bands, and regions containing free tracer at the gel front were excluded to improve the signal-to-background ratio. No additional bands were detected outside the displayed regions.

#### In vitro mouse plasma stability determination

The in vitro stability of the radiotracers in mouse plasma was evaluated by radio-HPLC over a period of 60 min. Approximately 370 kBq of formulated [^68^Ga]CREANT-101 or [^68^Ga]CREANT-102 were incubated with 100 µL of mouse plasma at 37 °C (300 rpm). Samples were collected after 30 and 60 min. At each timepoint, proteins were precipitated by adding 100 µL of ice-cold acetonitrile, followed by vortex mixing for 30 s and incubation on ice for 3 min. Samples were then centrifuged at 4000 rpm for 5 min at 4 °C. After centrifugation, the supernatant was separated from the pellet and transferred to a new vial. Subsequently, 60 µL of the recovered supernatant was diluted 1:1 with water and analyzed by radio-HPLC. The percentage of intact tracer was calculated as the ratio between the radioactivity peak corresponding to the intact compound and the total radioactivity detected during the run. Experiments were performed in duplicate for [^68^Ga]CREANT-101 and in triplicate for [^68^Ga]CREANT-102.

#### Cell culture

Five cancer cell lines (U-87 MG, HT-29, U266, K-562 and Jurkat T) and non-cancer cell line (HEK-293) were used. U-87 MG, HT-29 and HEK-293 were cultured in DMEM, while Jurkat T cells were maintained in RPMI-1640 medium. U266 and K-562 cells were cultured in IMDM. All culture media were supplemented with 10% fetal bovine serum (FBS), 2 mM L-glutamine, 1 mM sodium pyruvate, and 100 U/mL penicillin plus 100 µg/mL streptomycin (Life Technologies). Cells were maintained at 37 °C in a humidified atmosphere containing 5% CO₂.

#### Cell uptake

U-87 MG and HT-29 cells were seeded in 6-well plates at a density of 0.3 × 10^6^ cells per well (2 mL per well) and cultured as described in the previous section. After 48 h, approximately 370 kBq of the tracer [^68^Ga]CREANT-101 was added to each well and incubated for 1–2 h, with or without pre-incubation with K777 (5 µM).

For uptake determination, the culture medium was removed and collected, and the cells were washed twice with cold PBS (2 × 500 µL). The collected medium and PBS washes were combined to obtain the unbound fraction. Subsequently, cells were lysed by addition of 1 mL of 1 M NaOH per well to obtain the bound fraction. Radioactivity in both fractions was measured using an automatic γ-counter.

The results were quantified as a percentage of the total recovered activity, calculated as %ID (bound / (bound + unbound) × 100) per 10^6^ cells at 60 and 120 min, with and without preincubation of K777 at 60 min timepoint. Experiments were performed in triplicate for each condition.

#### Preparation of cell lysates

Upon reaching approximately 2 × 10^7^ cells, samples were centrifuged at 1000 × g for 2 min. The supernatant was discarded, and the cell pellet was washed twice with ice-cold PBS, followed by centrifugation at 1000 × g for 2 min. After removal of PBS, the pellet was then resuspended in 1 mL of cathepsin lysis buffer composed of 50 mM sodium citrate, 0.5% (w/v) CHAPS, 1% (v/v) NP-40, 2 mM DTT, pH 5. The lysates were incubated on ice for 10 min and then passed five times through a 29G needle and centrifuged at 7000 × g for 15 min at 4 °C. The supernatants were transferred to new tubes and total protein concentrations were quantified using a bicinchoninic acid (BCA) assay (Thermo Scientific).

#### Activity-based labeling of cell lysates and tumor homogenates

Protein aliquots (30 µg) obtained from either cell lysates or tumor homogenates were diluted to a final volume of 30 µL with cathepsin assay buffer. Samples were incubated with approximately 222 kBq of [^68^Ga]CREANT-101 at 37 °C for 30 min. Blocking experiments were performed by pre-incubating samples with K777 (1 µM) for 30 min before tracer addition. Subsequent processing and analysis were conducted according to the procedure described for recombinant cathepsin labeling.

#### Xenograft tumor models

Experimental procedures and protocols were performed following the revised European Directive 2010/63/EU on the protection of animals used for scientific purposes and were approved by the local ethical committee (2024-26, University of Antwerp, Belgium).

Animals were housed in individually ventilated cages under controlled environmental conditions including a 12 h light/dark cycle, temperature between 20 and 24 °C, and relative humidity ranging from 40% to 70%, with *ad libitum* access to food and water. Experimental groups were assigned by simple randomization.

U-87 MG and HT-29 cells were cultured as described above and used to establish subcutaneous xenograft models in female CD-1^−/−^ nude mice aged 6–8 weeks (body weight 20–25 g; Charles River Laboratories). Tumors were induced by injection into the hind limb using 6 × 10^6^ U-87 MG cells or 10 × 10^6^ HT-29 cells suspended in 100 µL sterile saline. Imaging experiments were initiated once tumor volumes reached approximately 200 mm^3^.

#### In vivo small-animal PET imaging

Dynamic PET imaging studies were performed in healthy female Balb/C mice and CD-1^−/−^ nude tumor-bearing mice aged 6–8 weeks and weighing 20–25 g. Mice were anesthetized with isoflurane (5% induction, 2% maintenance) and injected intravenously via the lateral tail vein. In all experiments, the injected mass of the radiotracer was approximately 1 µg. Healthy mice were injected with 4.3 ± 1.4 MBq (*N* = 10) of [^68^Ga]CREANT-101 and 3.3 ± 0.9 MBq (*N* = 10) of [^68^Ga]CREANT-102. Tumor-bearing mice were injected with [^68^Ga]CREANT-101 (6.5 ± 1.3 MBq (*N* = 4) for the U-87 model and 6.7 ± 0.7 MBq (*N* = 4) for the HT-29 model). Dynamic whole-body PET acquisitions were carried out for 60 min using an Inveon PET/CT scanner (Siemens) according to the following frame sequence: 12 × 10 s, 3 × 20 s, 3 × 30 s, 3 × 60 s, 3 × 150 s, and 9 × 300 s. Static whole-body PET scan was additionally acquired at 2 h 30 min post injection (p.i.) (1 × 1800 s frame). A whole-body CT scan was subsequently acquired for anatomical reference and segmentation. During the entire imaging procedure, body temperature was maintained using a heating pad.

Images were reconstructed using 8 iterations and 16 subsets with a 3D ordered subset expectation maximization (OSEM3D) algorithm, incorporating list-mode iterative reconstruction with proprietary spatially variant resolution modeling. Corrections for normalization, dead time, and CT-based attenuation were applied. PET images were reconstructed with a voxel size of 0.776 × 0.776 × 0.796 mm^3^ on a 128 × 128 × 159 grid.

Volumes of interest (VOIs) corresponding to organs and tissues including heart, lungs, liver, kidneys, bladder, muscle, brain, bone, and tumors were manually delineated on co-registered PET/CT images using PMOD software (version 3.6, PMOD Technologies, Zurich, Switzerland). Mean activity concentrations were extracted, and decay-corrected time–activity curves (TACs) were generated for each region. Tracer uptake was expressed as percentage injected dose per milliliter (%ID/mL = tissue uptake [kBq/mL]/injected dose [kBq] × 100).

At the conclusion of PET/CT acquisition, mice were euthanized by cervical dislocation and ex vivo biodistribution studies were performed as described in the corresponding section.

#### In vivo mouse plasma protein binding

To evaluate non-specific binding, approximately 185 kBq of radiotracer formulation was mixed with 500 µL MilliQ water and incubated for 3 min at room temperature. Three aliquots of 30 µL were collected for γ-counting prior to filtration. Subsequently, 300 µL of the mixture was transferred into a Centrifree ultrafiltration tube and centrifuged for 10 min at 2000 × g and 4 °C. Again, three aliquots of 30 µL of the ultrafiltrate were collected and radioactivity was measured using an automatic γ-counter. The unbound fraction was determined by dividing the mean CPM of the filtered fraction by that of the unfiltered samples.

For in vivo plasma protein binding (PPB) studies, at least 600 µL blood was collected in EDTA-coated tubes and maintained on ice. Plasma was isolated from blood samples by centrifugation at 7000 rpm for 5 min at 4 °C. From the resulting plasma, 500 µL was transferred into low-protein binding tubes, and three aliquots (30 µL) of unfiltered plasma were collected into a γ-counter tubes. Between 150 and 300 µL plasma was then loaded onto a Centrifree ultrafiltration tube and centrifuged immediately for 10 min at 2000 × g and 4 °C. Three aliquots (30 µL) of the filtrate were collected and all samples were then counted on an automatic γ-counter. The free fraction was calculated using the same equation applied for the non-specific binding assay and corrected by dividing it by the unbound fraction obtained from the non-specific binding control.

#### In vivo metabolite analysis

The metabolic stability of the tracers in vivo was assessed using plasma samples obtained from biodistribution studies at 30 and 60 min post injection (*N* = 3 per timepoint). Blood was collected in EDTA-containing tubes and plasma was isolated by centrifugation at 4000 rpm for 7 min at 4 °C. Samples were processed and analyzed following the same protocol described for the in vitro plasma stability experiments.

#### Ex vivo biodistribution studies

Biodistribution analyses were carried out in both healthy and tumor-bearing mice. Healthy female Balb/C mice (6–8 weeks old, 20–25 g) received intravenous injections of 4.3 ± 1.4 MBq of [^68^Ga]CREANT-101 or 3.3 ± 0.9 MBq of [^68^Ga]CREANT-102 in sterile 0.9% NaCl solution (*N* = 10 for each tracer). In all experiments, the injected mass of the radiotracer was approximately 1 µg. At 30, 60, or 180 min post injection (*N* = 3 at 30 and 60 min; *N* = 4 at 180 min), mice were anesthetized by inhalation of isoflurane/oxygen gas mixture (5% induction, 2% maintenance), and approximately 0.8 mL of blood was collected via cardiac puncture, followed immediately by cervical dislocation.

Organs and tissues including blood, heart, lungs, liver, spleen, pancreas, stomach, intestines, kidneys, bladder, urine, muscle, fat, bone, brain, and skin were excised, weighed, and counted using an automatic γ-counter (Wizard^2^ 2480, Revvity).

For tumor-bearing mice, U-87 MG and HT-29 xenograft models (*N* = 4/group) were injected with [^68^Ga]CREANT-101 (6.5 ± 1.3 MBq for the U-87 MG model and 6.7 ± 0.7 MBq for the HT-29 model) and euthanized 180 min after administration of [^68^Ga]CREANT-101. Radioactivity uptake in tissues was expressed as percentage injected dose per gram tissue (%ID/g).

#### Preparation of tumor homogenates

Following mice sacrifice, tumors were rapidly frozen in liquid nitrogen and stored at − 80 °C until processing. Frozen tissues were mechanically disrupted on dry ice using a mortar and pestle. The resulting material was transferred to Eppendorf tubes and resuspended in cathepsin lysis buffer at a ratio of 5µL of buffer per mg of tumor tissue. Samples were incubated on ice for 60 min, with intermittent resuspension every 15 min.

Homogenates were cleared by centrifugation at 12,000 × g for 10 min at 4 °C and the supernatants were collected into fresh tubes. Protein concentration was determined using a BCA assay (Thermo Scientific). Tumor homogenates were subsequently stored at − 80 °C until further analysis.

## Results

### Tracer design and synthesis

**Scheme 1:**
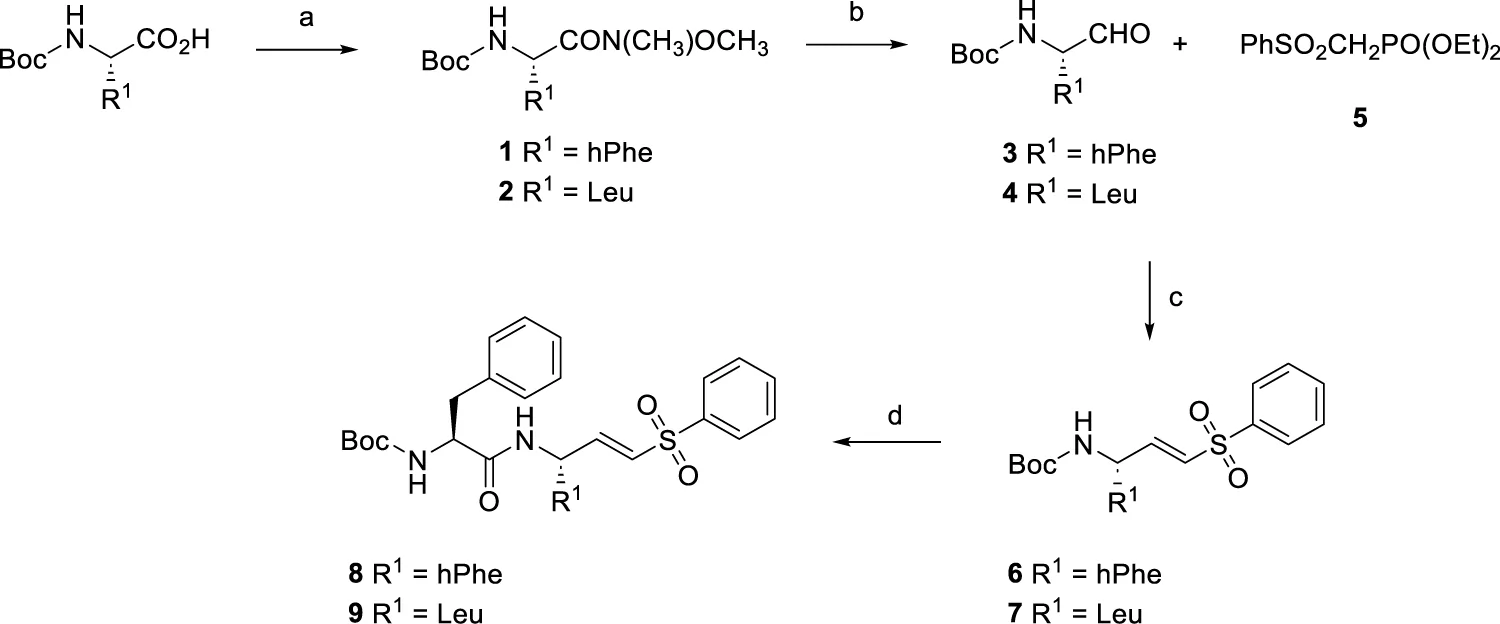
Reagents and conditions: (**a**) N, O-dimethylhydroxylamine hydrochloride, TEA, TBTU, HOBt, DCM, rt, 16 h, 99% (**b**) LiAlH_4_, THF, 0 °C, 1 h, 95%. (**c**) NaH, THF, 0 °C then rt, 3 h, (R_1_ = hPhe, 82%; R_1_ = Leu, 78%). (**d**) (1) TFA : DCM (1:1), rt, 2 h, quant. (2) Boc-AA, TEA, HOBt, TBTU, DMF, rt, 16 h, (R_1_ = hPhe, 85%; R_1_ = Leu, 88%)

The synthesis of the K777 scaffold and its P1-modified analogue followed the synthetic pathway developed by our research group (Capela et al. 2009; Oliveira et al. 2013), adapted from the method described by Palmer et al. (Palmer et al. 1995). To access both the P1 hPhe and the modified P1 Leu series, commercially available *N*-Boc-*L*-hPhe and *N*-Boc-*L*-Leu were independently converted into their corresponding Weinreb amides **1** and **2** by TBTU-catalyzed coupling to *N*,*O*-dimethylhydroxylamine hydrochloride and then reduced to the respective Boc-amino acid aldehydes **3** and **4** using LiAlH_4_ at 0 °C. Then a Horner-Wadsworth-Emmons (HWE) reaction between the aldehydes and the sulfone **5**, was performed affording the Boc-protected vinyl sulfones **6** and **7**. This reaction was highly stereoselective towards the *E*-isomer, which was confirmed by the high vicinal coupling constants in ^1^H-NMR (*J* = 15.0–15.2 Hz). Deprotection with TFA followed by coupling with and *N*-Boc-*L*-Phe using TBTU afforded dipeptidyl vinyl sulfones **8** and **9** (Scheme 1).

**Scheme 2:**
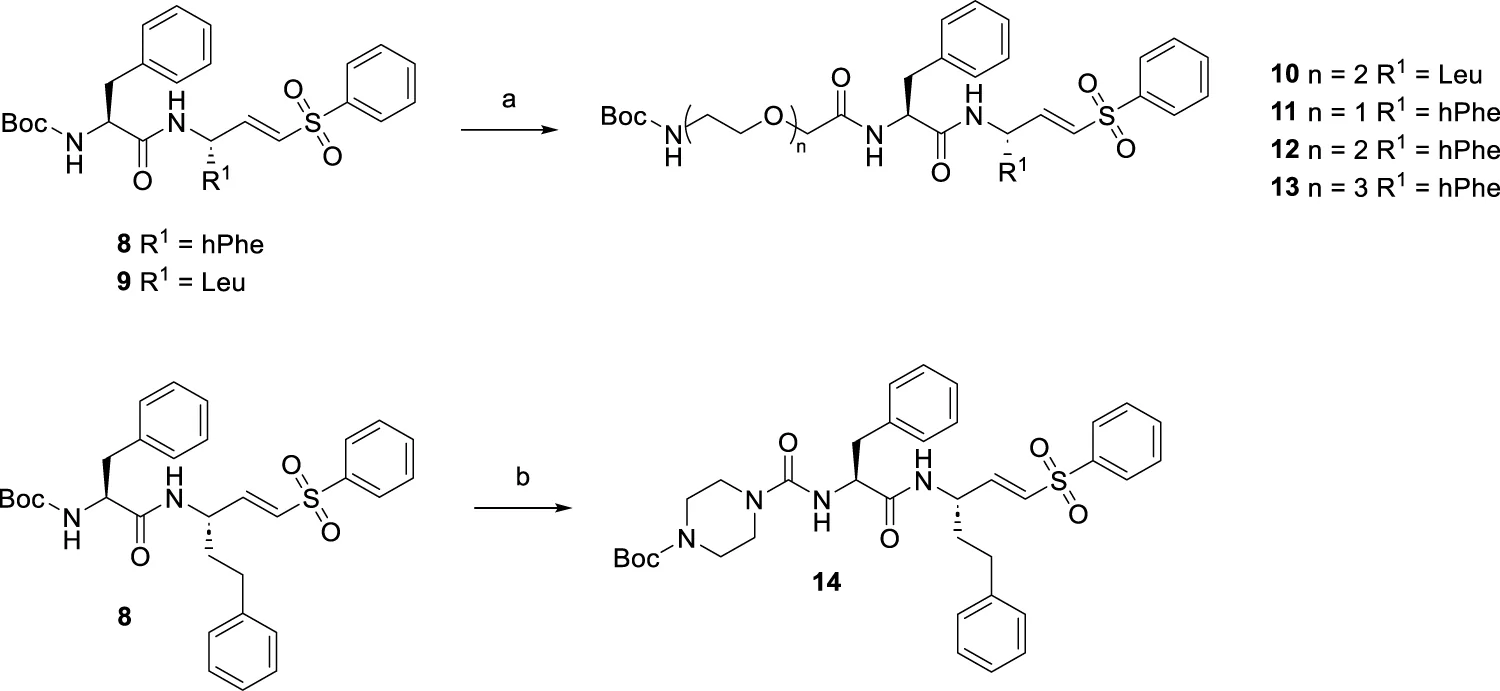
Reagents and conditions: (**a**) 1) TFA : DCM (1:1), rt, 2 h, quant. 2) Boc-NH-PEG-CH_2_COOH, DIPEA, TBTU, DMF, rt, 16 h, 70–96%. (**b**) (1) TFA : DCM (1:1), rt, 2 h, quant. (2) 4-boc-1-piperazinecarbonyl chloride, DIPEA, THF, -15 °C then rt, 16 h, 82%

Following TFA deprotection of the dipeptidyl vinyl sulfones, coupling with the terminal carboxylic acid of different N-Boc-protected PEG linkers was performed, resulting in the formation of compounds **10**–**13**. Similarly, deprotection of compound **8** followed by reaction with 4-Boc-1-piperazinecarbonyl chloride afforded compound **14**, incorporating a piperazine linker (Scheme 2).

**Scheme 3:**
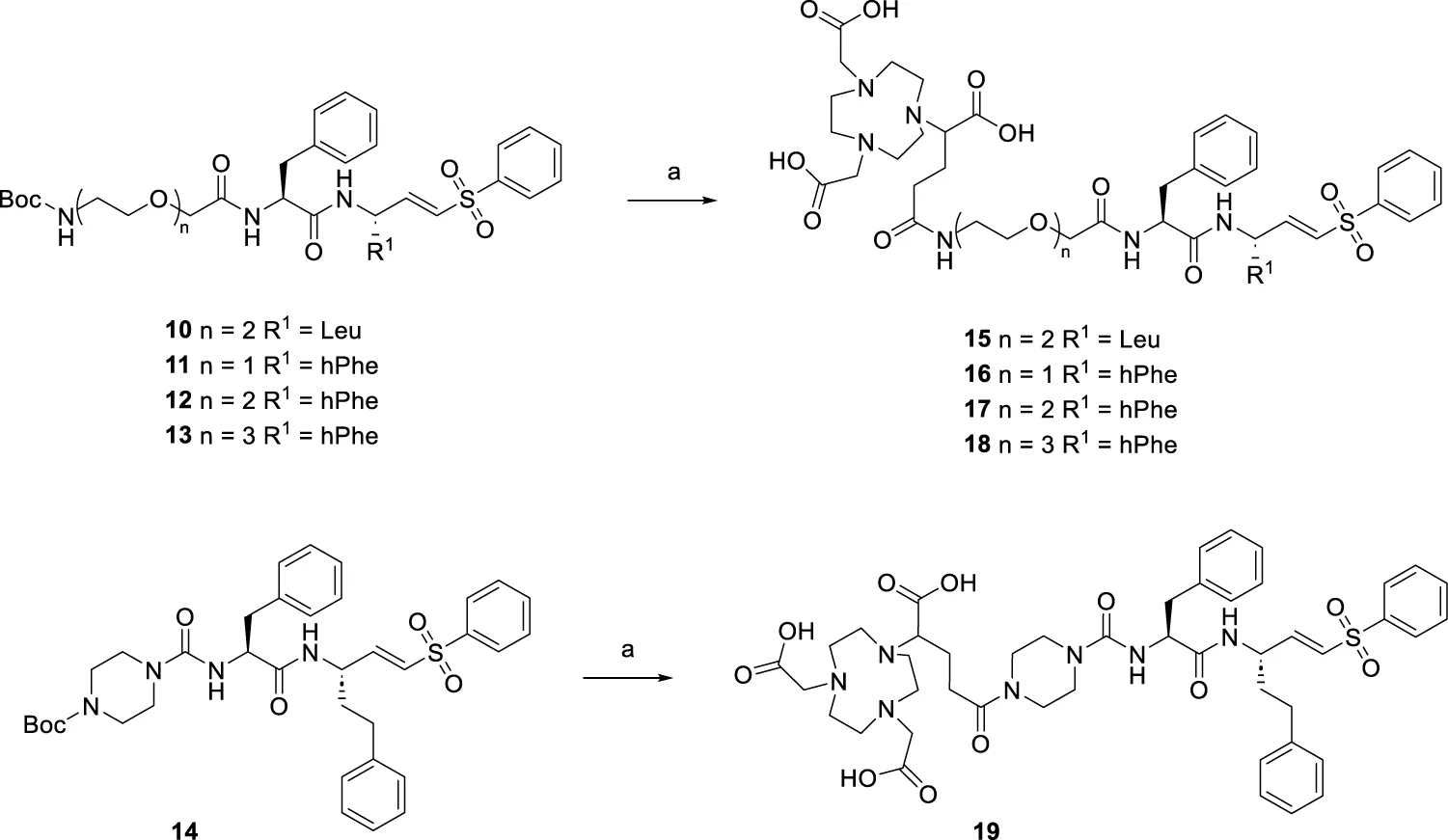
Reagents and conditions: a. 1) TFA : DCM (1:1), rt, 2 h, quant. 2) NODA-GA-NHS-ester, DIPEA, DMF, rt, 16 h, 28–50%

The conjugation with NODA-GA was carried out using the activated (*R*)-NODA-GA-NHS ester, which reacted with the free amine obtained after Boc deprotection, leading to the synthesis of the probe precursors **15**–**19** (Scheme 3).

**Scheme 4:**
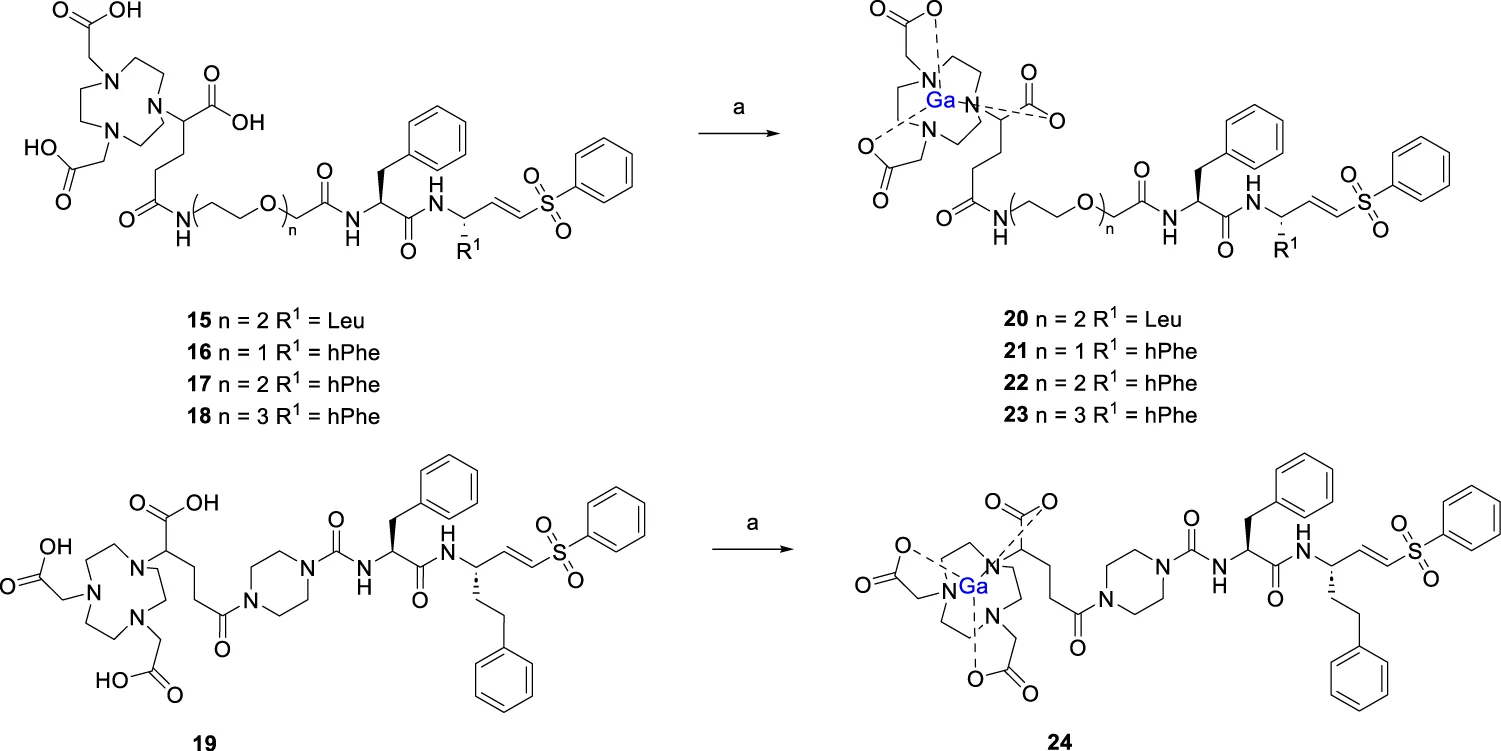
Reagents and conditions: a. ^nat^GaCl_3_, NH_4_OAc buffer (pH 5.5), 25 °C, 15 min., 93–97%

Finally, to obtain reference compounds of ^68^Ga-labeled probes, we synthesized the corresponding non-radiolabeled probe complexes using ^nat^GaCl_3_, obtaining compounds **20**–**24**, that were subsequently used for biochemical evaluation (Scheme 4).

#### Biochemical assays

The enzymatic characterization was carried out through fluorogenic assays designed to quantify the inhibitory activity and selectivity toward CatB and CatL of both the probe precursors (non-chelated NODA-GA form) and the reference compounds, corresponding to non-radiolabeled complexes (complexed with ^nat^Ga). For each inhibitor concentration the progress curves were individually fitted to extract the observed inactivation rate constant (*k*_obs_). These values were subsequently plotted against inhibitor concentration to calculate the maximum rate of inactivation (*k*_inact_) and the apparent inactivation constant (K_I_^app^). Finally, K_I_^app^ values were corrected for substrate competition using the Cheng-Prusoff equation with the experimentally determined K_m_ values for the substrate Z-FR-AMC (Supplemental Figure 1), getting the inactivation constant K_I_ and the inactivation efficiency *k*_inact_/K_I_ for each compound, listed in Table 1.

**Table 1:** Inhibition data of probe precursors and reference compounds for CatB and CatL

Compound	Chelator	Linker	P1	Cat B K_I_ [µM]	Cat L K_I_ [µM]	K_I_ SI (CatB/CatL)	Cat L k_inact_/K_I_ [M^− 1^ s^− 1^]
**17**	NODA-GA	PEG2	hPhe	> 100	0.32 ± 0.04	> 313	1.50 × 10^4^
**22**	[^nat^Ga] NODA-GA	PEG2	hPhe	> 100	0.32 ± 0.04	> 313	2.86 × 10^4^
**20**	[^nat^Ga] NODA-GA	PEG2	Leu	> 100	1.3 ± 0.2	> 77	5.43 × 10^3^
**23**	[^nat^Ga] NODA-GA	PEG3	hPhe	> 100	0.28 ± 0.03	> 357	1.92 × 10^4^
**21**	[^nat^Ga] NODA-GA	PEG1	hPhe	> 100	0.31 ± 0.04	> 322	2.61 × 10^4^
**19**	NODA-GA	Piperazine	hPhe	18 ± 1	0.32 ± 0.18	56	3.79 × 10^4^
**24**	[^nat^Ga] NODA-GA	Piperazine	hPhe	3.3 ± 1.1	0.11 ± 0.02	30	4.89 × 10^4^

The kinetic analysis of the compounds enabled the comparison of structural variations introduced in the P1 residue, the linker, and the presence or absence of ^nat^Ga-chelation showing their impact on enzyme inhibition and selectivity. Reference compound **22** (hPhe) displays a CatL K_I_ of 0.32 ± 0.04 µM with a CatB K_I_ > 100 µM (selectivity index, SI = K_I_ CatB / K_I_ CatL > 313), whereas the reference compound **20** (Leu) shows a reduced CatL potency (K_I_ = 1.3 ± 0.2 µM) and a lower selectivity index (SI > 77; CatB K_I_ > 100 µM). In our series of ^nat^Ga-chelated reference compounds, the different PEG linkers (**21**, **22** and **23**) showed very similar inhibitory potency toward CatL (K_I_ = 0.28–0.32 µM) and nearly identical selectivity indices. In contrast, compound **24** incorporating a piperazine linker exhibits a markedly different profile. Although the CatL K_I_ values remain in the sub-micromolar range (0.11 ± 0.02 µM), the CatB inhibition is substantially higher (K_I_ = 3.3 ± 1.1 µM), resulting in significantly lower selectivity indices (SI = 30) compared with PEG-linked compounds. As part of the study, the probe precursors were also evaluated to assess the impact of metal coordination on enzymatic inhibition. Thus, we compared pairs of precursors in which the only structural difference was the presence or absence of the metal. For PEG2-linked compounds, CatL inhibition remained essentially unchanged upon Ga^3+^ complexation (K_I_ = 0.32 µM for both precursor **17** and reference compound **22**) with identical selectivity indices (SI > 313), indicating that metal chelation does not influence CatL affinity or CatB/CatL selectivity, which provides a positive indication that the radiolabeled probe will bind to CatL selectively. The same observation applies to piperazine-linked compounds, where CatL inhibition and selectivity are practically unchanged between precursor (**19**, K_I_ = 0.32 µM ± 0.18, SI = 56) and reference compound (**24**, K_I_ = 0.11 ± 0.02 µM, SI = 30).

#### Radiochemistry

**Scheme 5:**
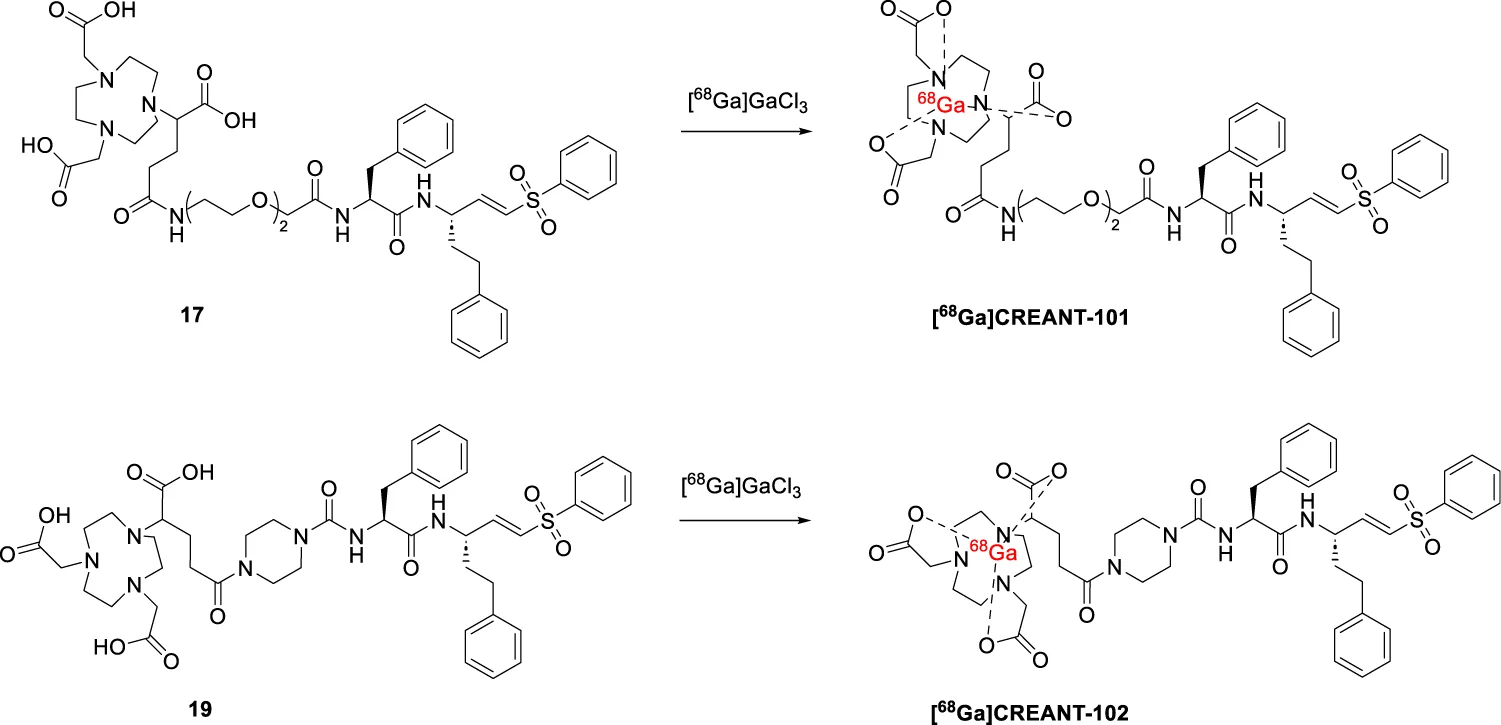
Schematic radiolabeling representation of precursors **17** and **19** with [^68^Ga]GaCl_3_ to yield [^68^Ga]CREANT-101 and [^68^Ga]CREANT-102, respectively. Detailed optimized conditions are summarized in Table 2

We therefore proceeded with radiolabeling two representative compounds (Scheme 5).

The PEG containing derivative **17** (radiolabeled as [^68^Ga]CREANT-101) was chosen as a representative of the PEG series, as all these compounds showed comparable selectivity toward CatL, with a K_I_ value of 0.32 µM. In parallel, compound **19** (radiolabeled as [^68^Ga]CREANT-102), bearing a piperazine moiety, was chosen, due to its superior potency toward CatL, with a K_I_ value of 0.11 µM. Radiolabeling conditions with [^68^Ga]GaCl_3_, were systematically optimized by varying temperature and reaction time.

Subsequently, different types of solid-phase extraction procedures (SPE) were evaluated for purification, to maximize radiochemical yield and purity. The final optimized conditions are summarized in Table 2, together with the corresponding radiochemical yields and purities.

**Table 2:** Optimization of [^68^Ga]GaCl_3_ radiolabeling conditions and purification procedures

Compound	Mass (µg)	Time (min)	Temperature (°C)	Purification (cartridge, mL EtOH)	RCC (%)	RCP (%)	RCY decay corrected (%)
[^68^Ga]CREANT-102	30	30	r.t.	-	70.45	n.d.	n.d.
[^68^Ga]CREANT-102	30	10	90	C8, 0.5	92.32	95.51	72
[^68^Ga]CREANT-102	50	30	r.t.	C8, 0.5	89.94	95.87	74
[^68^Ga]CREANT-102	50	10	90	C18, 0.5	91.88	95.23	69
**[** ^**68**^ **Ga]CREANT-102**	**50**	**5**	**80**	**tC2**,** 0.5**	**90.93 ± 0.90**	**95.21 ± 0.05**	**78**
[^68^Ga]CREANT-101	30	30	r.t.	-	75.23	n.d.	n.d.
[^68^Ga]CREANT-101	30	10	90	C8, 0.5	96.54	96.27	74
**[** ^**68**^ **Ga]CREANT-101**	**50**	**5**	**80**	**tC2**,** 0.5**	**94.11 ± 0.57**	**96.25 ± 0.47**	**87**

The table summarizes the different masses, temperatures, reaction times, and solid-phase extraction methods evaluated, along with the corresponding radiochemical conversions (RCC), radiochemical yields (RCY) and radiochemical purities (RCP). The final optimized conditions are highlighted in bold

**Fig. 2:**
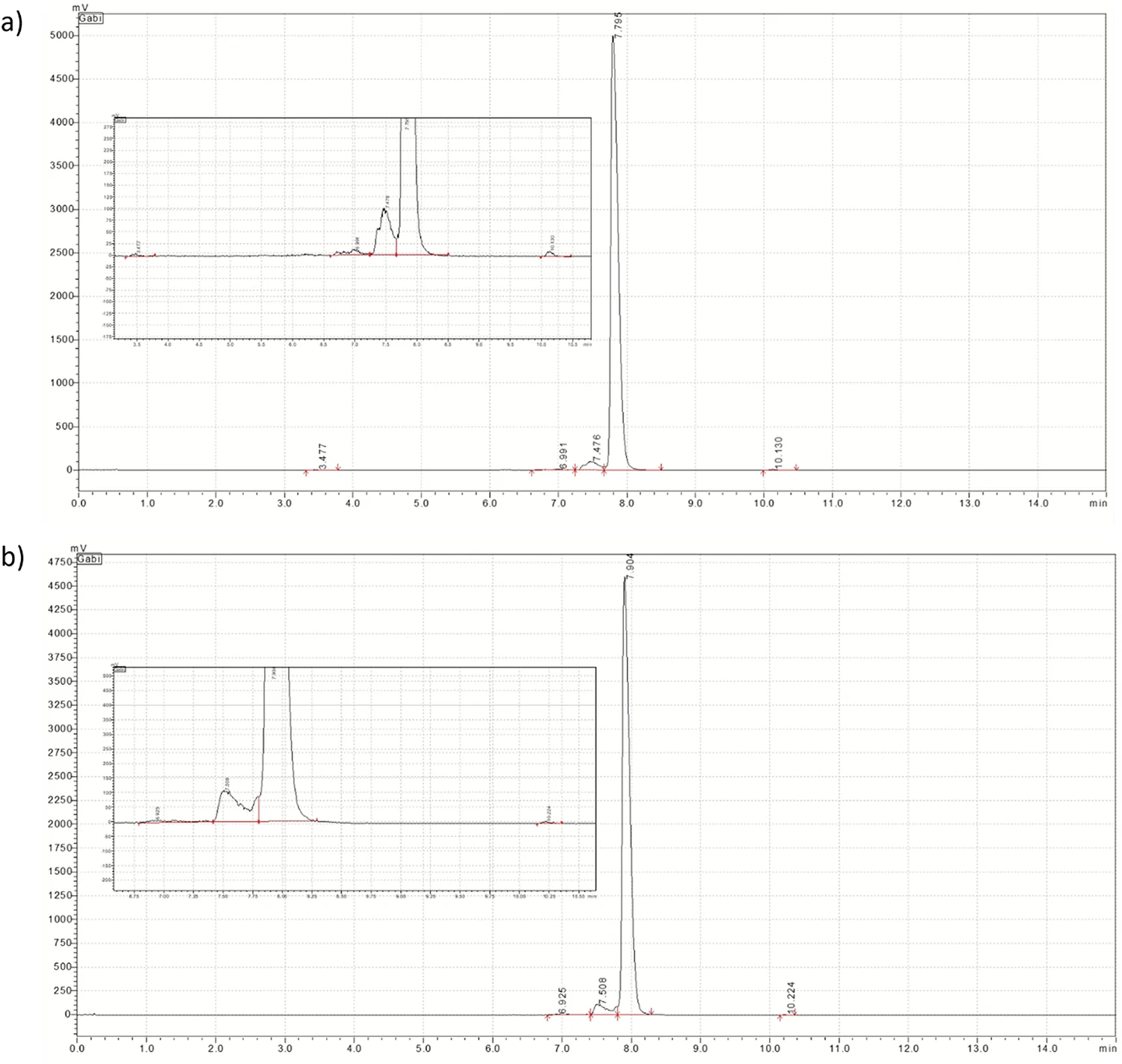
Radiochromatograms of [^68^Ga]CREANT-101 (**a**) and [^68^Ga]CREANT-102 (**b**)

Both radiotracers showed comparable yields, with slightly lower values for [^68^Ga]CREANT-102. Radiochromatograms consistently showed a RCP above 95%, with a main peak corresponding to the radiolabeled product (Fig. 2).

To confirm the identity of the radiolabeled products, the non-radioactive complexes **22** and **24** corresponding to [^68^Ga]CREANT 101 and [^68^Ga]CREANT 102, respectively, were analyzed in parallel on the same HPLC system (Supplemental Figures S7–S8).

The partition coefficients (logD values) of both [^68^Ga]CREANT-101 and [^68^Ga]CREANT-102 were determined. Both compounds were found to be slightly hydrophilic (logD= − 0.18 ± 0.01 and − 0.15 ± 0.01, respectively), showing that their specific linker architecture (PEG2 vs. piperazine) did not have a big impact in modulating lipophilicity.

### Activity‑based labeling of recombinant cathepsins

**Fig. 3:**
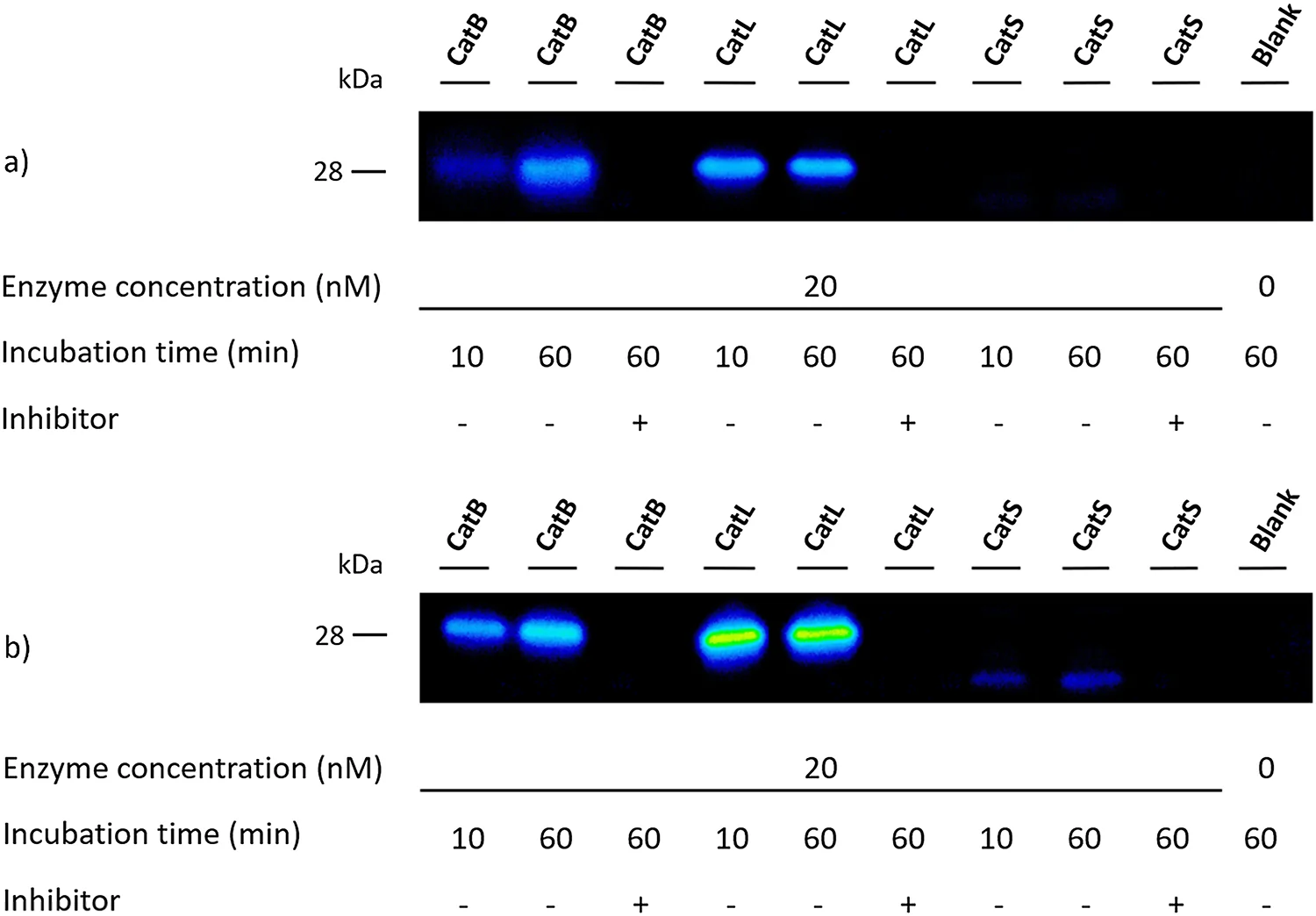
Biochemical evaluation of [^68^Ga]CREANT-101 (**a**) and [^68^Ga]CREANT-102 (**b**). For both (**a**) and (**b**), the images shown are composites obtained from cropped regions of separate gels. Gels for each respective compound were run and acquired in the same experiment under identical experimental conditions

Radioligand binding assays on recombinant CatB, CatL, and CatS were performed to evaluate the binding and selectivity of the radiolabeled ABPs for cathepsins. As expected, both [^68^Ga]CREANT-101 and [^68^Ga]CREANT-102 successfully recognized and bound CatL, demonstrated higher selectivity towards this target and confirming the results of the enzymatic assays (Fig. 3).

For each enzyme, the probes were incubated for 10–60 min at 37 °C, with efficient CatL labeling already observed at the earlier timepoint. In parallel, pre-incubation with K777 for 30 min before probe addition suppressed binding to both CatL and CatB, confirming target specificity and the broad inhibitory activity of K777 across cathepsin family members.

**Fig. 4:**
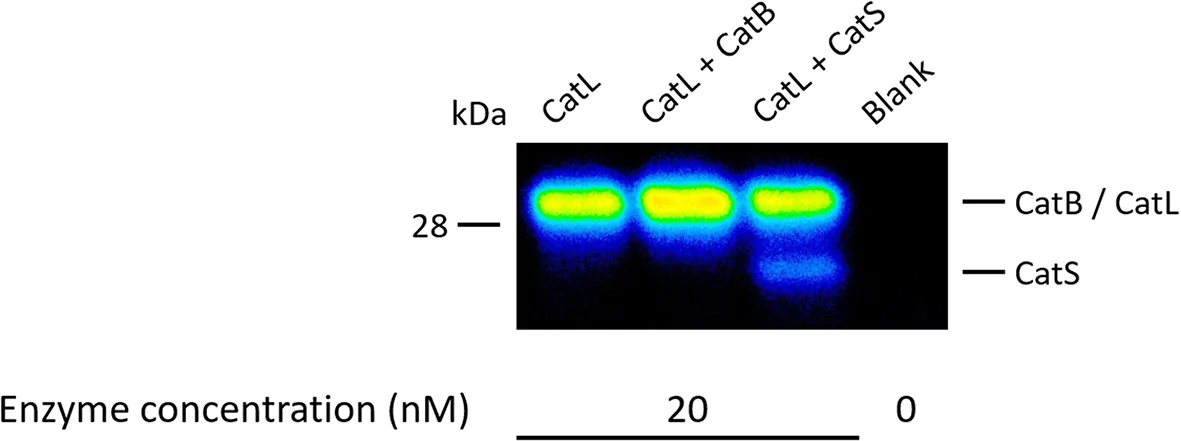
Competitive binding assay of [^68^Ga]CREANT-101 upon incubation with CatL alone or in co-incubation with CatB or CatS

Additionally, competitive binding assays were performed using recombinant CatB, CatL, and CatS to evaluate probe selectivity under co-incubation conditions. For these experiments, CatL was pre-mixed at equal concentrations with either CatB or CatS prior to probe incubation (Fig. 4).

Incubation of [^68^Ga]CREANT-101 with recombinant CatL and CatS resulted in two distinct bands, with a much more intense signal for CatL, confirming higher probe affinity toward this enzyme. Co-incubation with CatL and CatB produced a single band with slightly increased intensity relative to CatL alone, likely due to overlapping migration of the two enzymes.

### Stability

Tracers’ stability was assessed in formulation and PBS at RT over an 1 h. Both radiotracers showed high stability, retaining over 95% integrity in each condition.

Next, plasma stability was assessed to evaluate their suitability for in vivo application, incubating the radiotracers in mouse plasma and analyzed at 30 and 60 min. [^68^Ga]CREANT-101 demonstrated moderate stability, with a gradual decrease in intact tracer from 86% after 30 min to 73% at 60 min, while [^68^Ga]CREANT-102 showed higher stability, maintaining > 90% integrity at both time points.

**Fig. 5:**
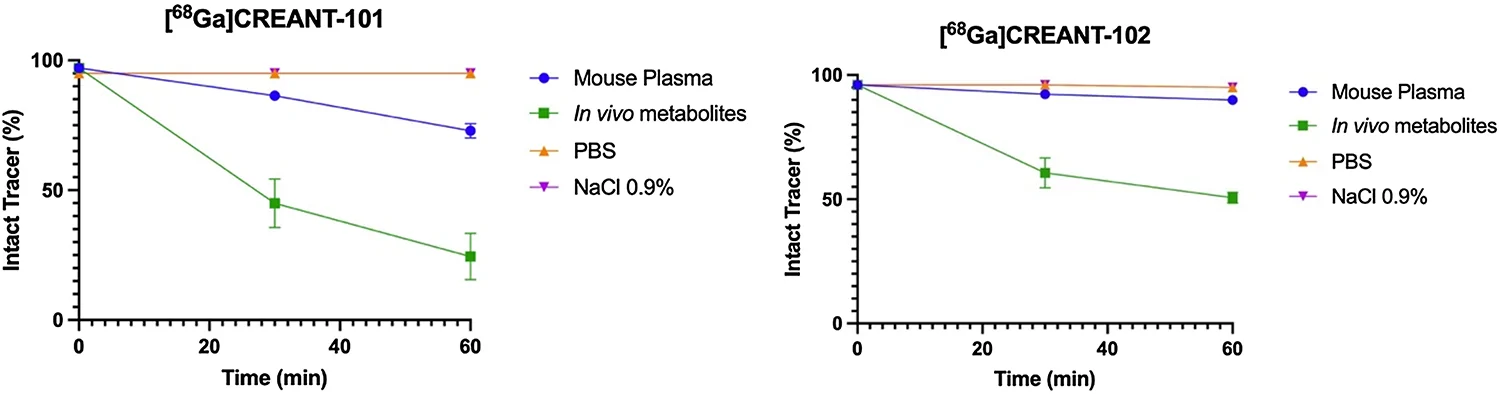
[^68^Ga]CREANT-101 and [^68^Ga]CREANT-102 stability in formulation, PBS, mouse plasma and in vivo Balb/C mice

In vivo metabolite analysis in Balb/C mice serum indicated a more rapid degradation for both the tracers. [^68^Ga]CREANT-101 underwent pronounced metabolic degradation, with 45.0 ± 9.3% intact tracer available at 30 min, decreasing to 24.5% ± 8.9 at 60 min. Conversely, [^68^Ga]CREANT-102 exhibited greater metabolic stability, with 60.6 ± 6.0% and 50.6 ± 2.2% of intact tracer remaining at 30 and 60 min, respectively, indicating a more favorable in vivo stability profile (Fig. 5).

## PET/CT imaging and biodistribution study

**Fig. 6:**
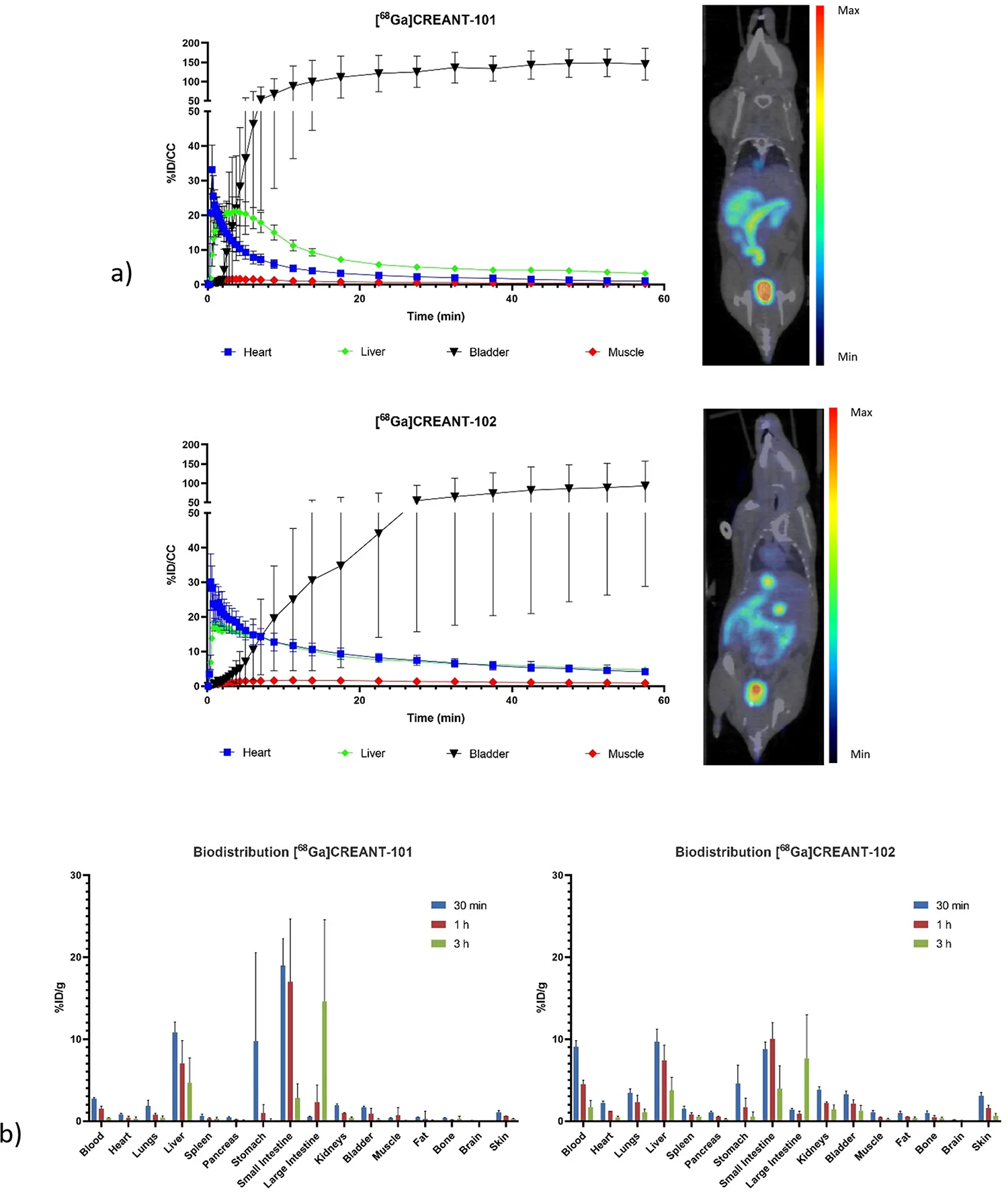
PET/CT imaging and ex vivo biodistribution. (**a**) Representative µPET/CT MIP images at 0–60 min post-injection and corresponding time–activity curves (TACs) of [^68^Ga]CREANT-101 and [^68^Ga]CREANT-102. (**b**) Ex vivo biodistribution analysis of the tracers

To characterize the pharmacokinetic properties of the radiotracers, in vivo µPET/CT imaging and ex vivo biodistribution studies were performed comparing both tracers in healthy Balb/C mice (Fig. 6).

Both tracers exhibited a mixed renal and hepatobiliary elimination pathway. Time-activity curves (TACs) revealed marked bladder accumulation for both tracers confirming significant renal clearance, although [^68^Ga]CREANT-102 accumulated more slowly than [^68^Ga]CREANT-101. Cardiac TACs further highlighted distinct pharmacokinetics between the tracers. [^68^Ga]CREANT-101 exhibited a rapid initial peak followed by fast washout, whereas [^68^Ga]CREANT-102 cleared more gradually, indicative of prolonged systemic circulation. Similar findings were observed in the ex vivo biodistribution data where [^68^Ga]CREANT-101 showed rapid blood clearance, decreasing from 2.71%ID/g at 30 min to 0.36%ID/g at 3 h and [^68^Ga]CREANT-102 displayed substantially higher blood activity at all time points (9.04%ID/g at 30 min, 4.50%ID/g at 1 h, and 1.71%ID/g at 3 h), consistent with slower systemic elimination. This behavior could be related to higher plasma protein binding observed for [^68^Ga]CREANT-102, likely contributing to its prolonged circulation, higher background activity, and increased non-specific tissue uptake. Specifically, [^68^Ga]CREANT-101 showed a higher free fraction (5.7% at 30 min and 9.5% at 3 h), whereas [^68^Ga]CREANT-102 exhibited markedly lower values (1.21% and 2.95%, respectively). In line with this findings, [^68^Ga]CREANT-101 displayed low uptake in most peripheral organs, including lungs (1.82 → 0.40%ID/g), heart (0.78 → 0.27%ID/g), and kidneys (1.94 → 0.40%ID/g) between 30 min and 3 h, while [^68^Ga]CREANT-102 showed higher uptake across these tissues, with similar trends observed in skin, muscle, pancreas, and spleen.

Hepatobiliary elimination was also evident for both tracers. [^68^Ga]CREANT-101 exhibited high early liver uptake (10.83%ID/g at 30 min, decreasing to 4.65%ID/g at 3 h) along with substantial small intestine activity at early time points (18.94%ID/g at 30 min), consistent with biliary excretion. [^68^Ga]CREANT-102 followed a similar pattern but with slower clearance, as indicated by more persistent intestinal activity (8.77%ID/g at 30 min and 3.97%ID/g at 3 h).

### Activity‑based labeling of cell lysates

**Fig. 7:**
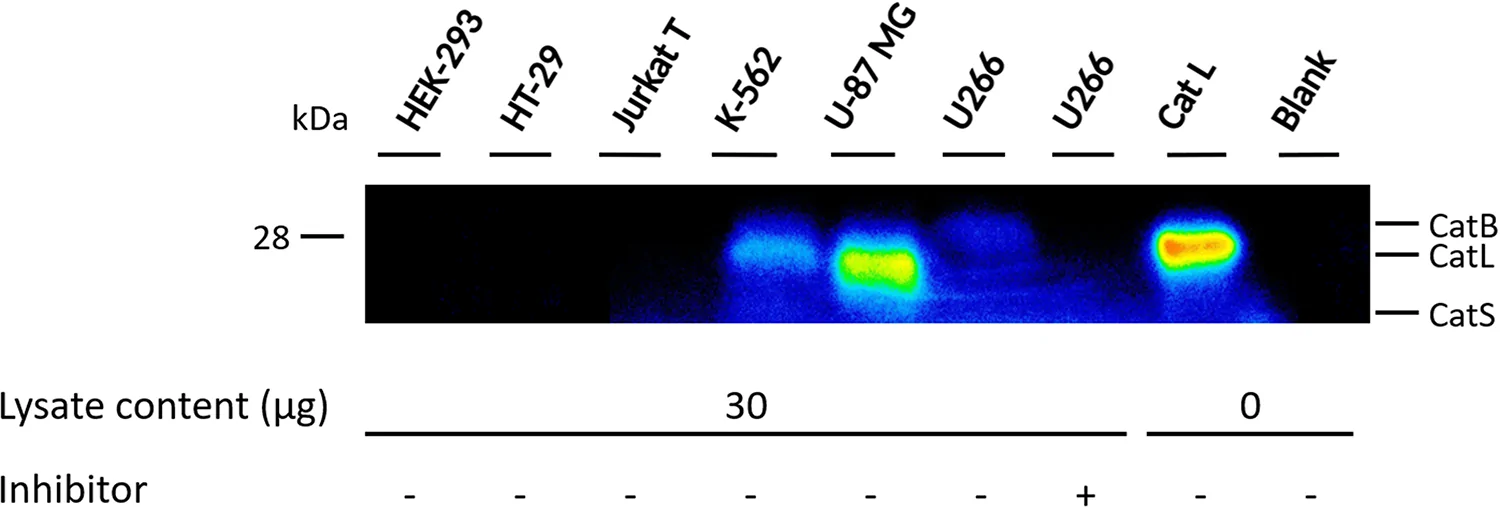
Binding profile of [^68^Ga]CREANT-101 in cell lysates. Molecular Weight (MW) of mature enzymes = CatB 30 kDa, CatL 28 kDa, CatS 24 kDa (Kirschke 2013; Mort 2013)

To select the cancer cell line with the highest CatL expression for the in vivo assessment of the probe, we investigated CatL expression in different human cancer cell lines. Based on recent literature and in-house availability, six human cancer cell lines were selected, including both expected positive and negative controls, respectively U-87 MG, HT-29, HEK-293, U266, K-562 and Jurkat T. [^68^Ga]CREANT-101 was incubated with lysates from the selected cell lines and analyzed by SDS-PAGE followed by autoradiography (Fig. 7).

To assess Cat signal specificity blocking experiments were performed using the U266 cell line, as strong CatL signal has been previously reported in this cell line (Poreba et al. 2019). In our experiment, the observed binding revealed stronger signal intensity in U-87 MG, also consistent with established literature documenting elevated CatL expression in this glioblastoma cell line (Kavčič et al. 2020; Primon et al. 2013). The preincubation of lysates with K777 significantly reduced signal intensity, thereby demonstrating selectivity of the probe. Based on these results, U-87 MG was selected as the positive cell lines for subsequent studies, while HT-29, which showed no detectable binding with the probe, was chosen as negative control.

### Cell uptake

**Fig. 8:**
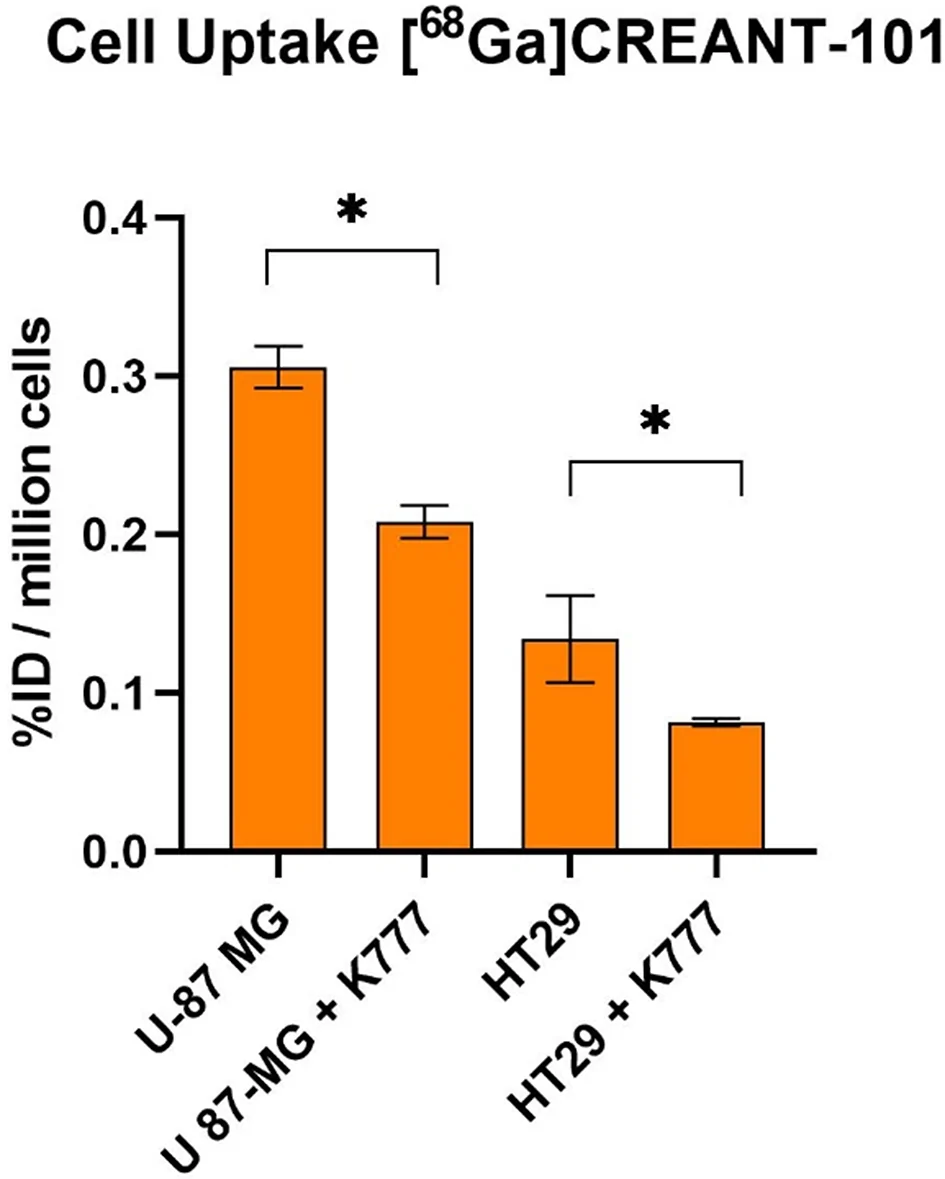
[^68^Ga]CREANT-101 uptake in U-87 MG and HT-29 cells at 60 min

To evaluate the cellular uptake of [^68^Ga]CREANT-101, an in vitro binding study was performed in intact U-87 MG and HT-29 cells (Fig. 8).

The results showed higher uptake in U-87 MG cells, reaching 0.31 ± 0.01%ID/10^6^ cells at 60 min and 0.32 ± 0.02%ID/10⁶ cells at 120 min, whereas HT-29 cells exhibited lower uptake values of 0.13 ± 0.03%ID/10^6^ at 60 min and 0.15 ± 0.01%ID/10^6^ at 120 min.

Preincubation with K777 led to a marked reduction in tracer uptake in both cell lines, decreasing to 0.21 ± 0.01%ID/10^6^ cells in U-87 MG (32% reduction; *p* = 0.0107) and 0.08 ± 0.002%ID/10^6^ cells in HT-29 (39% reduction; *p* = 0.0263).

Consistent with the known mechanism of vinyl sulfone-based inhibitors, which covalently and irreversibly target lysosomal cathepsins, both cell lines exhibited increasing tracer accumulation over time. The observed reduction in uptake upon K777 preincubation confirms that tracer binding is largely cathepsin-dependent.

#### PET/CT imaging in tumor-bearing mice

**Fig. 9:**
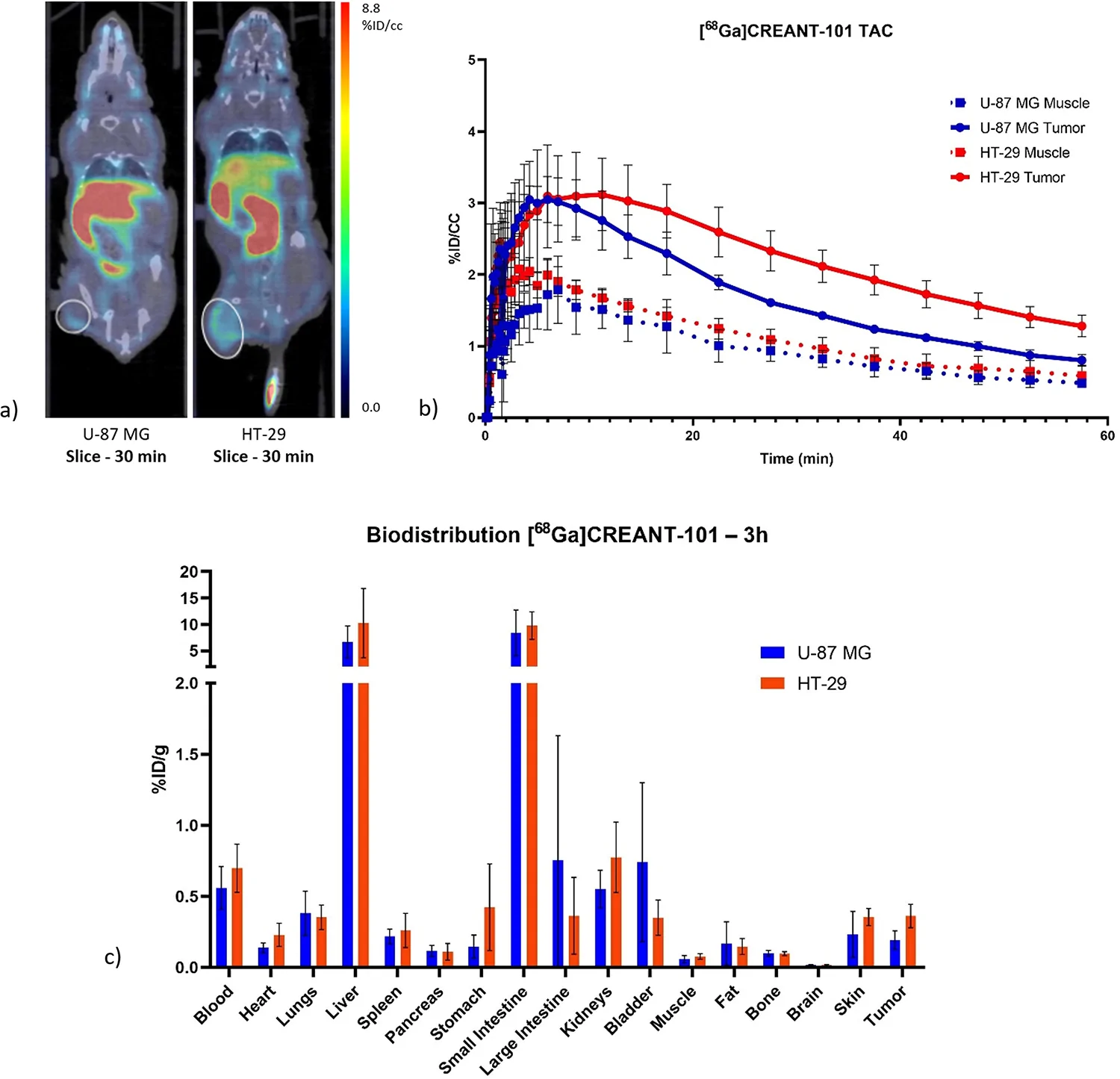
Representative evaluation of [^68^Ga]CREANT-101 in U-87 MG and HT-29 tumor-bearing mice. (**a**) PET image slices at 30 min p.i. (**b**) TACs. (**c**) Ex vivo biodistribution profile

Based on the tracer’s binding levels across the different tumor cell lines (Fig. 7), U-87 MG and HT-29 were selected as positive and negative xenograft models, respectively, in CD-1 nude mice. Analysis of time-activity curves revealed peak tumor uptake of 3.05 ± 0.32%ID/mL at 5.5 min in U-87 MG tumors and 3.12 ± 0.51%ID/mL at 10 min in HT-29 tumor (Fig. 9b). By the end of the dynamic acquisition (60 min p.i.), tumor-to-muscle ratios reached 1.67 ± 0.19 for U-87 MG and 2.28 ± 0.55 for HT-29.

Ex vivo biodistribution confirmed overall modest tumor uptake in both models, with values of 0.19 ± 0.06%ID/g for U-87 MG and 0.36 ± 0.08%ID/g for HT-29 at 3 h post-injection (Fig. 9c). These values corresponded to tumor-to-muscle ratios of 3.58 ± 0.93 and 5.15 ± 2.51, respectively. Importantly, minimal non-specific uptake was observed across most tissues, consistent with the low-background profile previously identified in healthy Balb/C mice. Minor differences were noted in intestinal uptake, suggesting slight variations in hepatobiliary clearance. In addition, high radioactivity levels in urine (12.02 ± 5.12%ID/g for U-87 MG and 14.18 ± 14.06%ID/g for HT-29) confirm a substantial contribution of renal excretion alongside hepatobiliary elimination.

Despite differences in CatL expression observed in vitro, no clear advantage in tracer uptake was observed for U-87 MG compared with HT-29 tumors (Fig. 9a). Analysis of the area under the curve (AUC) demonstrated a marked decrease from the early phase (0–40 min: 78.88 for U-87 MG; 95.86 for HT-29) to the late phase (40–60 min: 14.14 and 22.37, respectively), consistent with a predominant contribution of perfusion-driven uptake and limited tracer retention within the tumor. Nevertheless, the generally low background signal in non-target tissues (e.g., muscle and bone) supports a favorable imaging contrast profile despite the modest absolute tumor uptake.

#### Activity‑based labeling of tumor homogenates

**Fig. 10:**
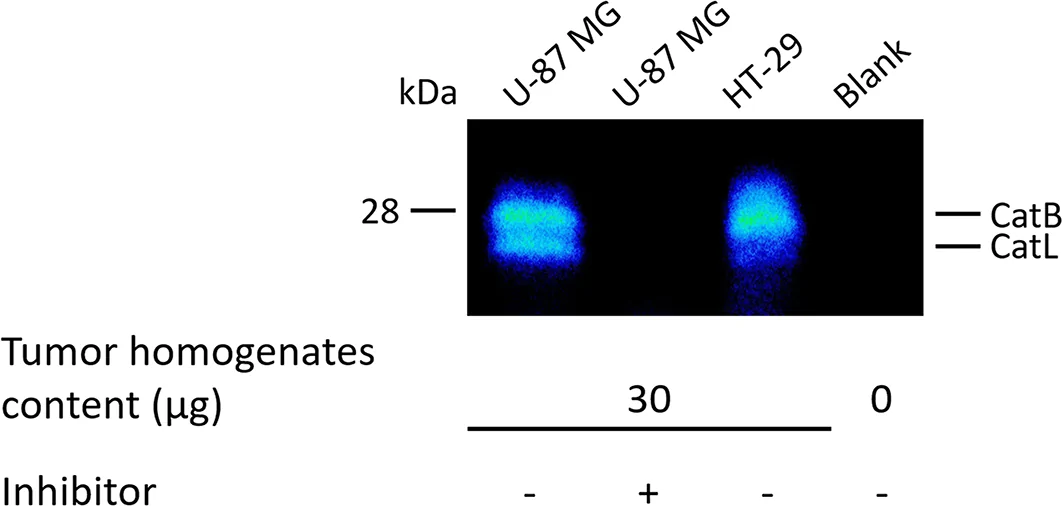
SDS-PAGE followed by ARG analysis of [^68^Ga]CREANT-101 incubated with tumor homogenates derived from U-87 MG and HT-29 cell lines

To determine whether the low probe accumulation at the tumor site was due to a failure in target engagement or unfavorable pharmacokinetic properties, we performed an ex vivo biochemical validation on tumor homogenates, revealing distinct radioactive bands in both the U-87 MG and the HT-29 tumor homogenates (Fig. 10), consistent with the molecular weights of mature CatL and CatB.

## Discussion

The synthesis of the various intermediates proceeded with generally good yields, in agreement with literature reports. The NODA-GA precursors were successfully purified using automated reversed-phase flash chromatography.

The analysis of inhibition data presented in Table 1, shows that retaining a hPhe at the P1 position enables both high potency and high selectivity against CatL, indicating that hPhe is the preferred P1 residue within this scaffold. In contrast, incorporating a Leu in the P1 position in the present series is associated with a reduced affinity against CatL. While previous studies show that a P1 Leu can yield highly potent CatL inhibitors, it must be considered that different electrophilic warheads inherently possess distinct reactivities and significantly impact the overall potency (Müller et al. 2023). The linker design proved to be a critical factor in modulating both potency and selectivity. The similar inhibitory potency observed among the different PEG linkers suggests that residues beyond the P3 position, and therefore variation in PEG length, does not significantly modulate binding to CatL. Conversely, the inhibition profile of the piperazine-linked compounds indicates that the piperazine linker increases CatB binding relative to CatL, resulting in reduced selectivity, whereas PEG linkers maintain strong CatL selectivity. No absolute selectivity threshold has been established for cathepsin-targeted agents, and different criteria have been adopted in the literature. Reported benchmarks range from SI > 10 for selective inhibition to approximately 100-fold differences for high selectivity between different cathepsins (Behring et al. 2023; Cianni et al. 2021). Within this range, the hPhe-containing PEG-linked compounds lie well above the values reported as indicative of high selectivity, while the piperazine-linked ones satisfies only the former. Overall, the kinetic data strongly suggest that PEG length has only a minor impact on potency and selectivity, whereas the linker scaffold (PEG vs. piperazine) substantially affects CatB/CatL selectivity, potentially through different interaction and positioning within the enzyme subsite S3 region. Importantly, the comparison between probe precursors and reference compounds indicate that Ga³⁺ chelation does not significantly affect binding affinity or selectivity, maintaining the selectivity profile of the precursors, which supported progression to biological studies.

The primary objective during the radiolabeling was to maximize radiochemical conversion (RCC) and yield (RCY) in the shortest possible time. This is a critical requirement given the short half-life of ^68^Ga, which ideally dictates synthesis times under 10 min (Davey and Paterson 2022). Although the NODA-GA chelator is known to allow ^68^Ga complexation at room temperature (Satpati et al. 2018), our preliminary tests required an increased precursor mass and longer reaction times to achieve yields comparable to those obtained with elevated temperatures and shorter incubations. We therefore proceed with the radiolabeling of probe precursors at higher temperatures. This approach aligns with the literature (Vatsa et al. 2019), where increasing the temperature during the radiolabeling of NODA-GA-conjugated compounds represents a key strategy to shorten reaction times. During purification, the use of a C18 cartridge led to excessive retention of the tracer which resulted in a loss of yield. Progressive reduction of retention of the stationary phase, from C8 and finally tC2 cartridges, improved product recovery and largely improved the final RCY.

As demonstrated by the radioligand binding assays, the probes showed high selectivity for CatL both when tested against individual enzymes and in co-incubation settings. Proteases of the cysteine cathepsin family can, in some combinations, degrade each other in a phenomenon known as “cathepsin cannibalism” (Barry and Platt 2012). This has been reported for specific pairs such as CatS and CatK, and it has been demonstrated that interactions among cathepsins can produce complex behaviors depending on incubation conditions and substrate availability. In particular, substrate presence may stabilize active enzymes and limit protease-on-protease degradation, while broader studies also describe cross-regulation or complementary activity with other proteases (Barry and Platt 2012; Ge et al. 2018; Parks et al. 2019; Pečar Fonović et al. 2024). To date there is no published evidence showing direct reciprocal degradation in vitro among the specific combination of cathepsins used in this study (Akkari et al. 2016; Di Spiezio et al. 2021). Considering that radioligand binding assays are performed using probe concentrations lower than enzyme levels, we cannot exclude the possibility of mutual degradation, as active proteases remain available for interaction. However, the results of the co-incubation experiment confirm selective recognition of CatL, with limited cross-reactivity toward CatB and CatS. These findings are consistent with the probe’s previously established affinity and selectivity for the individual cathepsins, as demonstrated through kinetic measurements and radioligand binding studies with each cathepsin tested separately.

Even though sharing similar lipophilicity, the two tracers had significantly different in vivo biodistribution profiles, mainly observed in blood retention, background activity, and clearance kinetics. Thus, even minor structural modification may significantly influence tracer behavior. [^68^Ga]CREANT-101 demonstrated more favorable pharmacokinetics, featuring rapid blood clearance, low background activity, and reduced non-specific tissue uptake, while [^68^Ga]CREANT-102 exhibited prolonged circulation, high blood retention, and showed extensive uptake, which might negatively affect image contrast and delay optimal imaging time points. Therefore, we selected [^68^Ga]CREANT-101 for further investigation, due to its potential to achieve higher tumor-to-background ratios. Yet, despite its favorable background profile, absolute tumor uptake in tumor-bearing mice, especially in U-87 MG xenografts, was lower than expected according to prior expression and enzyme engagement data. This result might be attributed to the tracer’s pharmacokinetic properties, including rapid systemic clearance, PPB, and progressive in vivo metabolism, which limit the amount of intact tracer available for covalent binding to extracellular and lysosomal cathepsins. Additionally, in vivo cellular compartmentalization may reduce the accessible fraction of active CatL compared to in vitro assays. An analogous limitation was previously reported for the first-generation activity-based probe ^64^Cu-Z-FK(DOTA)-AOMK which showed strong in vitro labeling but limited tumor accumulation (0.35%ID/g at 30 min p.i.) due to rapid clearance and off-target distribution. Further structural optimization, including addition of a bulky Cy5 fluorescent dye, substantially improved the probe’s *invivo* performance (Ren et al. 2011). As concerns PPB, [^68^Ga]CREANT-101 exhibits an increased free fraction over time. Although partially reversible binding has the potential to prolong circulation and enhance tumor uptake, this effect is likely constrained by the short half-life of ^68^Ga, resulting in radioactive decay before sufficient release and target engagement occur.

In vitro uptake studies also suggest limited cellular permeability which may restrict access to intracellular cathepsins. While cathepsins are actively secreted into the tumor microenvironment, this extracellular fraction represents only a minor portion of the total active enzyme pool (Biasizzo et al. 2022; Dana and Pathak 2020; Reiser et al. 2010; Yadati et al. 2020; Zhang et al. 2025). Moreover, when the probes are evaluated in cell lysates or tumor homogenates, they are exposed to a concentrated pool of cathepsins that does not reflect the physiological conditions encountered in intact cells or living tissues, where extracellular enzyme concentrations are substantially lower.

The moderate uptake and partial blocking observed in HT-29 cells are consistent with the detectable expression of active cysteine cathepsins in this cell line. Therefore, HT-29 should be regarded as a low CatL expressing rather than a negative model. Accordingly, tracer uptake in HT-29 cells reached approximately 40% of that measured in U-87 MG cells, while K777 produced a comparable relative reduction in tracer uptake in both cell lines. In addition, the covalent, time-dependent mechanism of vinyl sulfone-based probes enables progressive accumulation of probe-labeled enzyme during the incubation period, even when active enzyme levels are low. A minor contribution from CatB also cannot be excluded due to the residual cross-reactivity of the probes. The residual uptake observed following K777 treatment is most likely attributable to passive diffusion, and non-specific binding.

Taken together, these findings suggest that the combination of limited cellular permeability and relatively low intracellular concentration of active target enzyme restricts access to the principal pool of active cathepsins, thereby limiting tracer accumulation in tumors in vivo.

The labeling pattern observed in ex vivo tumor homogenates differed from the single-band profile detected in U-87 MG lysates in vitro and from the absence of signal expected in HT-29 samples. HT-29 was selected as a negative control based on undetectable binding and reported low CatL expression (Esfahani et al. 2014; Poreba et al. 2019), thus the target engagement in this xenograft model was unexpected. However, cathepsin expression can vary markedly between in vitro systems and in vivo tumor tissues. In HT-29 cells, for instance, active CatB expression has been shown to increase depending on differentiation state (Stefanis et al. 1997). Importantly, these differences in expression are not solely intrinsic to cancer cells but are also strongly influenced by other cell types that infiltrate the tumor. Tumor-associated cells, such as fibroblasts, neutrophils, endothelial cells, and tumor-associated macrophages (TAMs), robustly overexpress cysteine cathepsins heavily contributing to the overall protease activity in the tissue (Mohamed and Sloane 2006; Olson and Joyce 2015). Consistently, co-culture of HT-29 cells with fibroblasts and monocytic cells has been reported to induce a 5- to 30-fold increase in CatB levels compared with standard monolayers (Krueger et al. 2005). Such context-dependent variations in protease abundance likely contribute to the unexpected signals and their intensities. Notably, the use of xenograft models may underestimate immune-derived protease activity, highlighting the need for validation in immunocompetent systems. Partial labeling of CatB also cannot be excluded, consistent with the modest cross-reactivity observed in vitro with recombinant cathepsins and the probe’s time-dependent inhibition mechanism. A relevant analogous example of this phenomenon was observed with the probe BMV083, which showed almost exclusive selectivity for CatS over CatB in vitro in cultured cells; when tested in tumor homogenates, CatB emerged as a prominent secondary target, as it was the most active and abundant cysteine cathepsin in the specific tumor model used (Verdoes et al. 2012). Likewise, the CatL-selective probe MP-cL3 was shown to exhibit cross-reactivity with CatB upon prolonged exposure in cellular systems (Poreba et al. 2018).

Through this ex vivo biochemical validation, we confirmed the covalent engagement of the probe within the tissue environment, labeling active cathepsins. Importantly, pre-treatment with K777 resulted in an absence of signal in the U-87 MG tumor homogenate, proving the specificity of the binding. Therefore, while a similar blocking experiment would be necessary to confirm the specificity of the signal in HT-29 tissues, we can conclude with these results that the probe can successfully bind its functional targets, suggesting that the limiting factor for in vivo imaging was probably driven by insufficient bioavailability at the tumor site, reduced cell uptake, and restricted enzyme accessibility rather than a lack of target affinity.

## Conclusions

In this study, we successfully developed and preclinically evaluated a novel class of ⁶⁸Ga-labeled PET ABPs targeting CatL for non-invasive tumor imaging. By utilizing the validated K777 vinyl sulfone scaffold, our SAR investigations demonstrated that retaining a hPhe residue at the P1 position is preferred over Leu to achieve high CatL potency and selectivity. Furthermore, exploring modifications at the P3 position, revealed that PEG linkers maintain strong CatL selectivity, while the introduction of a piperazine linker increased off-target binding to CatB. Comparing the kinetic parameters between probe precursors and reference compounds we also confirmed that gallium chelation do not alter the probe’s enzymatic affinity and selectivity profile. In vivo profiling of two radiotracers, the PEG2-linked [^68^Ga]CREANT-101 and the piperazine-linked [^68^Ga]CREANT-102, highlighted the profound impact of linker architecture on pharmacokinetics. Although [^68^Ga]CREANT-102 displayed greater metabolic stability, it showed elevated blood retention and high non-specific background tissue uptake while [^68^Ga]CREANT-101 exhibited a highly favorable imaging profile characterized by rapid systemic clearance and low background activity. Despite presenting excellent selectivity and clearance, [^68^Ga]CREANT-101’s in vivo PET imaging in U-87 MG and HT-29 tumor-bearing mice showed only modest overall tumor uptake. Subsequent ex vivo biochemical validation on tumor homogenates proved that the probe successfully and specifically binds with active cathepsins within the target tissue. This crucial finding indicates that the limiting factor for optimal in vivo imaging was not a lack of target affinity, but rather insufficient tumor bioavailability driven by the rapid systemic clearance and in vivo metabolism of the tracer, with limited cellular internalization representing an additional limitation. Therefore, this work provides a solid basis for the rational development of selective CatL-targeted ABPs and emphasizes that future investigations must prioritize the optimization of pharmacokinetic properties to exploit their full diagnostic utility in precision oncology.

## Supplementary Information

Below is the link to the electronic supplementary material.

Supplementary Material 1

## Data Availability

The datasets used and/or analysed during the current study are available from the corresponding author on reasonable request.

## Abbreviations

ABPs: Activity-based probes
ARG: Autoradiography
AUC: Area under the curve
BCA: Bicinchoninic acid
CatB: Cathepsin B
CatL: Cathepsin L
CatK: Cathepsin K
CatS: Cathepsin S
CHAPS: 3-[(3-cholamidopropyl)dimethylammonio]-1-propanesulfonate
CPM: Counts per minute
CT: Computed tomography
DCM: Dichloromethane
DIPEA: N, N-diisopropylethylamine
DMF: Dimethylformamide
DMSO: Dimethyl sulfoxide
DOTA: 1,4,7,10-tetraazacyclododecane-1,4,7,10-tetraacetic acid
DTT: Dithiothreitol
ECM: Extracellular matrix
EDTA: Ethylenediaminetetraacetic acid
ESI: Electrospray ionization
FBS: Fetal bovine serum
HOBt: Hydroxybenzotriazole
HPLC: High performance liquid chromatography
HRMS: High resolution mass spectrometry
K_M_: Michaelis-Menten constant
k_*inact*_: Maximum rate of inactivation
k_*inact*_/K_I_: Inactivation efficiency
K_I_^app^: Apparent inactivation constant
k_*obs*_: Observed inactivation rate constant
K_I_: Inactivation constant
LRMS: Low resolution mass spectrometry
MIP: Maximum intensity projection
NHS: N-hydroxysuccinimide
NMR: Nuclear magnetic resonance
NODA-GA: 1,4,7-triazacyclononane-1-glutaric acid-4,7-acetic acid
NOTA: 1,4,7-triazacyclononane-1,4,7-triacetic acid
NP-40: Nonidet P-40
PBS: Phosphate buffered saline
PEG: Polyethylene glycol
PET: Positron emission tomography
PPB: Plasma protein binding
RCC: Radiochemical conversion
RCP: Radiochemical purity
RCY: Radiochemical yield
SAR: Structure–activity relationship
SDS-PAGE: Sodium dodecyl sulfate polyacrylamide gel electrophoresis
SI: Selectivity index
SPE: Solid-phase extraction
TACs: Time-activity curves
TBTU: O-(benzotriazol-1-yl)-N, N,N’,N’-tetramethyluronium tetrafluoroborate
TFA: Trifluoroacetic acid
THF: Tetrahydrofuran
TME: Tumor microenvironment
v_0_: Initial velocity
v_s_: Steady-state velocity
VOIs: Volumes of interest
^68^Ga: Gallium-68
^nat^Ga: Natural Gallium
