# Supplementary material for: Development and preclinical evaluation of selective vinyl sulfone-based probes for PET imaging of tumor-associated cathepsins
Source: EJNMMI Radiopharm Chem. 2026 Aug 31;11:64. doi: 10.1186/s41181-026-00493-5 (PMC13550339; doi:10.1186/s41181-026-00493-5)
Supplement: Supplementary file 1 [file 41181_2026_493_MOESM1_ESM.docx]

Development and Preclinical Evaluation of Selective Vinyl Sulfone-Based Probes for PET Imaging of Tumor-Associated Cathepsins

Oliviero Cini^1,2^, Alan Miranda^2^, Daan Willocx^2^, Angeliki Karakasidi^2^, Anke de Groot^2^, Thales do Valle Moreira^2,3^, Ana P. Leandro^1^, Maria M. M. Santos^1^, Rui Moreira^1^ and Filipe Elvas^2^

1) Research Institute for Medicines (iMed.ULisboa), Faculty of Pharmacy, Universidade de Lisboa, Av. Prof. Gama Pinto, 1649-003 Lisboa, Portugal;
2) Molecular Imaging and Radiology, University of Antwerp, Universiteitsplein 1, 2610 Wilrijk, 2610 Antwerpen, Belgium;
3) Institute of Pharmacy and Biochemistry, University of Mainz, Staudingerweg 5, 55128 Mainz, Germany.

Corresponding Author: Filipe Elvas, Molecular Imaging Center Antwerp, University of Antwerp, Universiteitsplein 1, 2610 Wilrijk, Belgium.

Email: [filipe.elvas@uantwerpen.be](mailto:filipe.elvas@uantwerpen.be)

# Supporting Information

## Biochemical assay and Km determination

The experimental setup for the enzymatic assays was defined by integrating elements from two published procedures. The buffer composition was adapted from Ren et al. (Ren et al. 2013), which provided the most suitable conditions for enzyme stability and catalytic performance of the different cathepsins, while the assay execution followed the protocol established by Jung et al. (Jung et al. 2021) selected for its validated kinetic workflow and results analysis. Kinetic parameters of CatB and CatL were experimentally determined, in order to ensure accurate calculation of inactivation constants of the inhibitors considering the substrate-competition for irreversible covalent inhibitors. For both CatB and CatL, the Michaelis–Menten constant (K_m_) was obtained testing different concentrations of the fluorogenic substrate Z-FR-AMC (Figure S1).

Supplemental Figure 1. Michaelis-Menten plot of Z-FR-AMC for Km determination of CatB (a) and CatL (b).

## General procedure for the synthesis of Weinreb Amides

A solution of Boc-AA-OH (5.37 mmol, 1.0 mol. equiv.) in dichloromethane (12 mL) was treated with triethylamine (18.79 mmol, 3.5 mol. equiv.), TBTU (5.91 mmol, 1.1 mol. equiv.), and HOBt (5.91 mmol, 1.1 mol. equiv.). The resulting suspension was stirred until complete dissolution of all the components (around 15 minutes), after which N,O-dimethylhydroxylamine (5.91 mmol, 1.1 mol. equiv.) was added. The reaction mixture was stirred overnight (16h) at room temperature under a nitrogen atmosphere. Subsequently, the mixture was diluted with dichloromethane (50 mL) and washed successively with 3 N hydrochloric acid (50 mL), saturated sodium bicarbonate solution (50 mL), and brine (50 mL). The organic phase was dried over sodium sulfate, filtered, and concentrated to afford the Weinreb amide intermediate. This procedure was performed according to a previously publish synthetic protocol (Capela et al. 2009).

***tert*-butyl (S)-(1-(methoxy(methyl)amino)-1-oxo-4-phenylbutan-2-yl)carbamate - 1**

Colorless oil (1.71 g, 99% yield). ^1^H NMR (300 MHz, CDCl_3_) δ = 7.34 – 7.24 (m, 2H), 7.23 – 7.15 (m, 3H), 5.22 (d, *J* = 9.4 Hz, 1H), 4.76 – 4.59 (m, 1H), 3.62 (s, 3H), 3.16 (s, 3H), 2.77 – 2.57 (m, 2H,), 2.11 – 1.93 (m, 1H), 1.92 – 1.71 (m, 1H), 1.45 (s, 9H) ppm. The NMR data are in good agreement with those reported in the literature (Roush et al. 1998).

***tert*-butyl (S)-(1-(methoxy(methyl)amino)-4-methyl-1-oxopentan-2-yl)carbamate - 2**

Colorless oil (587 mg, 99% yield). ^1^H NMR (300 MHz, CDCl_3_) δ = 5.04 (d, *J* = 9.6 Hz, 1H), 4.71 (m, 1H), 3.78 (s, 3H), 3.19 (s, 3H), 1.81 – 1.61 (m, 2H), 1.45 – 1.40 (m, 10H), 0.96 (d, *J* = 6.5 Hz, 3H), 0.92 (d, *J* = 6.7 Hz, 3H) ppm. The NMR data are in good agreement with those reported in the literature (Fernández-de-la-Pradilla et al. 2023)

## General procedure for the synthesis of aldehydes

The Weinreb amide **1** or **2** (5.37 mmol, 1.0 mol. equiv.) was dissolved in dry tetrahydrofuran (24 mL) and cooled to 0 °C. Lithium aluminium hydride (5.91 mmol, 1.1 mol. equiv.) was added dropwise, and the resulting suspension was stirred at 0 °C for 1 h under a nitrogen atmosphere. The excess hydride was then carefully quenched with an aqueous solution of potassium hydrogenosulfate (9.39 mmol, 1.75 mol. equiv.). The reaction mixture was extracted with diethyl ether (3 × 30 mL), and the combined organic layers were washed successively with 3 N hydrochloric acid (45 mL), saturated sodium bicarbonate solution (45 mL), and brine (45 mL). The organic phase was dried over sodium sulfate, filtered, and concentrated to afford the aldehyde in pure form. This procedure was performed according to a previously publish synthetic protocol (Capela et al. 2009).

**tert-butyl (S)-(1-oxo-4-phenylbutan-2-yl)carbamate - 3**

Colorless oil (1.34 g, 95% yield). ^1^H NMR (300 MHz, CDCl_3_) *δ* = 9.55 (s, 1H), 7.35 – 7.12 (m, 5H), 5.06 (br s, 1H), 4.36 – 4.18 (m, 1H), 2.77 – 2.63 (m, 2H), 2.31 – 2.14 (m, 1H), 1.97 – 1.82 (m, 1H), 1.47 (s, 9H) ppm. The NMR data are in good agreement with those reported in the literature (Roush et al. 1998).

**tert-butyl (S)-(4-methyl-1-oxopentan-2-yl)carbamate - 4**

Colorless oil (442 mg, 95% yield). ^1^H NMR (300 MHz, CDCl_3_) *δ* = 9.58 (s, 1H), 4.92 (br s, 1H), 4.29 – 4.17 (m, 1H), 1.81 – 1.55 (m, 2H), 1.48 – 1.30 (m, 10H), 0.97 (d, *J* = 6.5 Hz, 3H), 0.96 (d, *J* = 6.5 Hz, 3H) ppm. The NMR data are in good agreement with those reported in the literature (Fernández-de-la-Pradilla et al. 2023).

## General procedure for the synthesis of sulfones

A solution of the thiophosphonate (5.76 mmol, 1 mol. equiv.) in acetic acid (8 mL) was heated to 50 °C, and 30% hydrogen peroxide (21.17 mmol, 3.5 mol. equiv.) was added dropwise while maintaining the temperature below 80 °C. The reaction mixture was then stirred at 85 °C for 2 h 30 min. After cooling to room temperature, the mixture was diluted with ice water (30 mL), and the pH was adjusted to 8–9 by the dropwise addition of 10 M sodium hydroxide solution. The aqueous phase was extracted with dichloromethane (3 x 45 mL) and washed with 1 M sodium hydrogenosulfite solution (45 mL). The organic layer was dried over sodium sulfate, filtered, and concentrated under reduced pressure to afford the pure compound. This procedure was performed according to a previously publish synthetic protocol (Capela et al. 2009).

**diethyl ((phenylsulfonyl)methyl)phosphonate - 5**

Colorless oil (1.34 g, 80% yield). ^1^H NMR (300 MHz, CDCl_3_) *δ* = 8.05 - 7.94 (m, 2H), 7.71 – 7.62 (m, 1H), 7.62 – 7.51 (m, 2H), 4.22 – 4.08 (m, 4H), 3.76 (d, *J* = 16.8 Hz, 2H), 1.29 (t, *J* = 7.1 Hz, 6H) ppm. The NMR data are in good agreement with those reported in the literature (Fuchs et al. 2023).

## General procedure for the synthesis of vinyl sulfones

A solution of the sulfone (0.54 mmol, 1.2 mol. equiv.) in dry tetrahydrofuran (3 mL) was cooled to −10 °C, and sodium hydride (0.58 mmol, 1.3 mol. equiv.) was added. After 10 min, a precooled solution of the appropriate aldehyde (0.45 mol. mmol, 1.0 equiv.) in dry tetrahydrofuran (3 mL) was added. The reaction mixture was allowed to warm to room temperature and stirred for 3 h under a nitrogen atmosphere. The mixture was then diluted with diethyl ether (20 mL) and washed with brine (2 × 20 mL). The organic phase was dried over sodium sulfate, filtered, and concentrated. Purification by flash chromatography afforded the desired compound. This procedure was performed according to a previously publish synthetic protocol (Capela et al. 2009).

**tert-butyl (S,E)-(5-phenyl-1-(phenylsulfonyl)pent-1-en-3-yl)carbamate - 6**

White solid (715 mg, 82% yield). ^1^H NMR (300 MHz, CDCl_3_) *δ* = 7.91 – 7.84 (m, 2H), 7.65 – 7.58 (m, 1H), 7.56 – 7.49 (m, 2H), 7.33 – 7.10 (m, 5H), 6.89 (dd, *J* = 15.0, 5.1 Hz, 1H), 6.42 (dd, *J* = 15.1 , 1.5 Hz, 1H), 4.53 (d, *J* = 8.6 Hz, 1H), 4.42 – 4.27 (m, 1H), 2.78 – 2.58 (m, 2H), 2.00 – 1.74 (m, 2H), 1.39 (s, 9H) ppm. The NMR data are in good agreement with those reported in the literature (Roush et al. 1998).

**tert-butyl (S,E)-(5-methyl-1-(phenylsulfonyl)hex-1-en-3-yl)carbamate - 7**

White solid (125 mg, 78% yield). ^1^H NMR (300 MHz, CDCl_3_) *δ* = 7.92 – 7.82 (m, 2H), 7.67 – 7.47 (m, 3H), 6.87 (dd, *J* = 15.1, 4.6 Hz, 1H), 6.42 (d, *J* = 15.1, 1H), 4.50 – 4.26 (m, 2H), 1.76 – 1.59 (m, 1H), 1.46 – 1.28 (m, 11H), 0.93 (d, *J* = 6.6 Hz, 6H) ppm.

## General procedure for the synthesis of dipeptidyl vinyl sulfones

A solution of Boc-protected vinyl sulfone (1.03 mmol, 1.0 mol. equiv.) in dichloromethane (10 mL) and trifluoroacetic acid (10 mL) was stirred at room temperature for 3 h. The solvent was then removed under reduced pressure, and the residue was kept under high vacuum until further use.

Separately, a solution of the appropriate Boc-AA-OH (1.13 mmol, 1.1 mol. equiv.) in dry dimethylformamide (14 mL) was cooled to 0 °C under a nitrogen atmosphere. HOBt (1.24 mmol, 1.2 mol. equiv.), TBTU (1.24 mmol, 1.2 mol. equiv.), and triethylamine (3.61 mmol, 3.5 mol. equiv.) were added, and the mixture was stirred at 0 °C for 30 min until complete dissolution. A solution of the previously deprotected vinyl sulfone in dry dimethylformamide (14 mL) was then added. The reaction mixture was allowed to warm to room temperature and stirred overnight under nitrogen. The reaction was diluted with ethyl acetate (90 mL) and washed successively with 1 N hydrochloric acid (2 × 45 mL), saturated sodium bicarbonate solution (2 × 45 mL), and brine (2 × 45 mL). The organic layer was dried over sodium sulfate, filtered, and concentrated. Purification by flash column chromatography afforded the dipeptidyl vinyl sulfone in pure form. This procedure was performed according to a previously publish synthetic protocol (Capela et al. 2009).

**tert-butyl ((S)-1-oxo-3-phenyl-1-(((S,E)-5-phenyl-1-(phenylsulfonyl)pent-1-en-3-yl)amino)propan-2-yl)carbamate - 8**

White solid (487 mg, 85% yield). ^1^H NMR (300 MHz, CDCl_3_) *δ* = 7.89 – 7.82 (m, 2H), 7.70 – 7.61 (m, 1H), 7.61 – 7.53 (m, 2H), 7.33 – 7.06 (m, 10H), 6.81 (dd, *J* = 15.1, 4.5 Hz, 1H), 6.07 (d, *J* = 15.1 Hz, 1H), 5.84 (d, *J* = 8.7 Hz, 1H), 4.89 (br s, 1H), 4.75 – 4.63 (m, 1H), 4.30 – 4.17 (m, 1H), 3.02 (d, *J* = 7.1 Hz, 2H), 2.71 – 2.50 (m, 2H), 2.03 – 1.71 (m, 2H), 1.43 (s, 9H) ppm. LRMS *m/z* (ESI^+^): 549 [M+H]^+^. The NMR data are in good agreement with those reported in the literature (Roush et al. 1998).

**tert-butyl ((S)-1-(((S,E)-5-methyl-1-(phenylsulfonyl)hex-1-en-3-yl)amino)-1-oxo-3-phenylpropan-2-yl)carbamate - 9**

White solid (138 mg, 88% yield). ^1^H NMR (300 MHz, CDCl_3_) *δ* = 7.87 – 7.81 (m, 2H), 7.67 – 7.59 (m, 1H), 7.58 – 7.50 (m, 2H), 7.28 – 7.10 (m, 5H), 6.77 (dd, *J* = 15.1, 4.6 Hz, 1H), 6.07 (d, *J* = 15.1 Hz, 1H), 5.78 (d, *J* = 8.6 Hz, 1H), 4.94 (s, 1H), 4.76 – 4.64 (m, 1H), 4.29 – 4.17 (m, 1H), 3.06 – 2.96 (m, 2H), 1.59 – 1.46 (m, 1H), 1.44 – 1.30 (m, 11H), 0.88 (d, *J* = 6.5 Hz, 3H), 0.87 (d, *J* = 6.6 Hz, 3H) ppm. LRMS *m/z* (ESI^+^): 501 [M+H]^+^.

## General procedure for the conjugation of dipeptidyl vinyl sulfones with different linkers

A solution of Boc–dipeptidyl vinyl sulfone (0.10 mmol, 1 mol. equiv.) in dichloromethane (2 mL) and trifluoroacetic acid (2 mL) was stirred at room temperature for 3 h. The solvent was then removed under reduced pressure, and the residue was kept under high vacuum until further use.

**PEG Linkers**: Separately, a solution of the appropriate linker (0.12 mmol, 1.2 mol. equiv.) in dry dimethylformamide (5 mL) was prepared under a nitrogen atmosphere. TBTU (0.12 mmol, 1.2 mol. equiv.) and N,N-diisopropylethylamine (0.36 mmol, 3 mol. equiv.) were added, and the mixture was stirred until complete dissolution. A solution of the previously deprotected dipeptidyl vinyl sulfone in dry dimethylformamide (5 mL) was then added, and the reaction mixture was stirred overnight under nitrogen. The reaction mixture was diluted with ethyl acetate (50 mL) and washed successively with 1 N hydrochloric acid (2 × 25 mL), saturated sodium bicarbonate (2 × 25 mL), and brine (2 × 25 mL). The organic layer was dried over sodium sulfate, filtered, and concentrated. Purification by preparative TLC afforded the desired compound in pure form.

**Piperazine linker**: The resulting deprotected vinyl sulfone was dissolved in dry tetrahydrofuran (2 mL), and N,N-diisopropylethylamine (0.33 mmol, 3.0 mol. equiv.) was added. The mixture was cooled to −15 °C under a nitrogen atmosphere, and once fully dissolved, 4-boc-1-piperazinecarbonyl chloride (0.13 mmol, 1.1 mol. equiv.) was added. The reaction mixture was stirred at −15 °C for 15 min, then allowed to warm to room temperature and stirred overnight under nitrogen. The reaction mixture was diluted with water (25 mL) and extracted with ethyl acetate (3 × 25 mL). The combined organic layers were washed with water (3 × 25 mL) and brine (2 × 25 mL), dried over sodium sulfate, filtered, and concentrated. Purification by preparative TLC afforded the desired compound in pure form.

**tert-butyl ((10S,13S)-10-benzyl-15-methyl-8,11-dioxo-13-((E)-2-(phenylsulfonyl)vinyl)-3,6-dioxa-9,12-diazahexadecyl)carbamate - 10**

Yellow oil (54 mg, 88% yield). ^1^H NMR (300 MHz, CDCl_3_) *δ* = 7.89 – 7.78 (m, 2H), 7.67 – 7.59 (m, 1H), 7.59 – 7.50 (m, 2H), 7.42 (d, *J* = 8.3 Hz, 1H), 7.24 – 7.10 (m, 5H), 6.72 (dd, *J* = 15.1, 5.0 Hz, 1H), 6.08 (d, *J* = 8.7 Hz, 1H), 6.00 (d, *J* = 15.0 Hz, 1H), 5.51 (br s, 1H), 4.72 – 4.54 (m, 2H), 4.01 – 3.89 (m, 2H), 3.61 – 3.48 (m, 6H), 3.39 – 3.23 (m, 2H), 3.06 – 2.99 (m, 2H), 1.61 – 1.47 (m, 2H), 1.43 (s, 9H), 1.38 – 1.30 (m, 1H), 0.86 (d, *J* = 6.5 Hz, 6H) ppm. LRMS *m/z* (ESI^+^): 646 [M+H]^+^.

**tert-butyl (2-(2-oxo-2-(((S)-1-oxo-3-phenyl-1-(((S,E)-5-phenyl-1-(phenylsulfonyl)pent-1-en-3-yl)amino)propan-2-yl)amino)ethoxy)ethyl)carbamate - 11**

White solid (56 mg, 96% yield). ^1^H NMR (300 MHz, CDCl_3_) *δ* = 7.89 – 7.79 (m, 2H), 7.68 – 7.60 (m, 1H), 7.59 – 7.51 (m, 2H), 7.29 – 7.00 (m, 10H), 6.75 (dd, *J* = 15.1, 4.9 Hz, 1H), 6.23 (d, *J* = 8.4 Hz, 1H), 6.02 (dd, *J* = 15.1, 1.7 Hz, 1H), 4.97 – 4.86 (m, 1H), 4.66 – 4.52 (m, 2H), 3.90 (s, 2H), 3.52 – 3.43 (m, 2H), 3.35 – 3.25 (m, 2H), 3.10 – 3.02 (m, 2H), 2.61 – 2.49 (m, 2H), 1.94 – 1.68 (m, 2H), 1.46 (s, 9H) ppm. LRMS *m/z* (ESI^+^): 550 [M+H - Boc]^+^.

**tert-butyl ((10S,13S,E)-10-benzyl-8,11-dioxo-13-phenethyl-15-(phenylsulfonyl)-3,6-dioxa-9,12-diazapentadec-14-en-1-yl)carbamate - 12**

Yellow oil (60 mg, 70% yield). ^1^H NMR (300 MHz, CDCl_3_) *δ* = 7.87 – 7.79 (m, 2H), 7.70 – 7.59 (m, 1H), 7.61 – 7.50 (m, 2H), 7.45 (d, *J* = 7.8 Hz, 1H), 7.30 – 7.11 (m, 8H), 7.10 – 7.01 (m, 2H), 6.74 (dd, *J* = 15.1, 4.9 Hz, 1H), 6.17 (d, *J* = 7.2 Hz, 1H), 5.96 (d, *J* = 15.3 Hz, 1H), 5.53 (br s, 1H), 4.68 – 4.54 (m, 2H), 3.96 (d, 1H), 3.94 (d, 1H), 3.62 – 3.48 (m, 6H), 3.41 – 3.23 (m, 2H), 3.08 – 2.98 (m, 2H), 2.60 – 2.46 (m, 2H), 1.93 – 1.69 (m, 2H), 1.42 (s, 9H) ppm. LRMS *m/z* (ESI^+^): 694 [M+H]^+^.

**tert-butyl ((13S,16S,E)-13-benzyl-11,14-dioxo-16-phenethyl-18-(phenylsulfonyl)-3,6,9-trioxa-12,15-diazaoctadec-17-en-1-yl)carbamate – 13**

Yellow oil (50 mg, 75% yield). ^1^H NMR (300 MHz, CDCl_3_) *δ* = 7.87 – 7.80 (m, 2H), 7.67 – 7.60 (m, 1H), 7.59 – 7.51 (m, 2H), 7.44 (d, *J* = 7.8 Hz, 1H), 7.29 – 7.14 (m, 8H), 7.09 – 7.04 (m, 2H), 6.76 (dd, *J* = 15.1, 4.9 Hz, 1H), 6.36 – 6.24 (m, 1H), 6.02 (dd, *J* = 15.1, 1.8 Hz, 1H), 5.13 (br s, 1H), 4.67 – 4.50 (m, 2H), 3.98 (s, 2H), 3.64 – 3.57 (m, 8H), 3.54 – 3.49 (m, 2H), 3.34 – 3.21 (m, 2H), 3.17 – 2.98 (m, 2H), 2.64 – 2.47 (m, 2H), 1.95 – 1.70 (m, 2H), 1.42 (s, 9H) ppm. LRMS *m/z* (ESI^-^): 737 [M+H]^-^.

**tert-butyl 4-(((S)-1-oxo-3-phenyl-1-(((S,E)-5-phenyl-1-(phenylsulfonyl)pent-1-en-3-yl)amino)propan-2-yl)carbamoyl)piperazine-1-carboxylate - 14**

White solid (58 mg, 82% yield). ^1^H NMR (300 MHz, CDCl_3_) *δ* = 7.89 – 7.79 (m, 2H), 7.69 – 7.49 (m, 3H), 7.30 – 7.09 (m, 8H), 7.10 – 7.01 (m, 2H), 6.77 (dd, *J* = 15.1, 4.7 Hz, 1H), 6.32 (d, *J* = 8.5 Hz, 1H), 6.06 (dd, *J* = 15.1, 1.7 Hz, 1H), 4.98 (d, *J* = 7.3 Hz, 1H), 4.65 – 4.55 (m, 1H), 4.50 – 4.37 (m, 1H), 3.44 – 3.18 (m, 8H), 3.02 (d, *J* = 7.4 Hz, 2H), 2.65 – 2.46 (m, 2H), 1.95 – 1.70 (m, 2H), 1.45 (s, 9H). LRMS *m/z* (ESI^+^): 662 [M+H]^+^.

## RP-HPLC chromatograms of the non-radioactive ^nat^Ga complexes

Supplemental Figure 2. RP-HPLC chromatogram of non-radioactive ^nat^Ga complex **20** (t_R_ = 12.743 min, 95.46% purity by UV area).

Supplemental Figure 3. RP-HPLC chromatogram of non-radioactive ^nat^Ga complex **21** (t_R_ = 12.950 min, 95.21% purity by UV area).

Supplemental Figure 4. RP-HPLC chromatogram of non-radioactive ^nat^Ga complex **22** (t_R_ = 13.130 min, 97.70% purity by UV area).

Supplemental Figure 5. RP-HPLC chromatogram of non-radioactive ^nat^Ga complex **23** (t_R_ = 13.057 min, 95.12% purity by UV area).

Supplemental Figure 6. RP-HPLC chromatogram of non-radioactive ^nat^Ga complex **24** (t_R_ = 13.147 min, 100.00% purity by UV area).

## Comparative HPLC analysis of [^68^Ga]CREANT-101 and [^68^Ga]CREANT-102 with non-radioactive complexes

Supplemental Figure 7. RP-HPLC chromatogram (upper) of non-radioactive complex **22** and radio-HPLC chromatogram (lower) of [^68^Ga]CREANT-101.

Supplemental Figure 8. RP-HPLC chromatogram (upper) of non-radioactive complex **22** and radio-HPLC chromatogram (lower) of [^68^Ga]CREANT-102.

## References SI

Capela R, Oliveira R, Gonçalves LM, Domingos A, Gut J, Rosenthal PJ, et al. Artemisinin-dipeptidyl vinyl sulfone hybrid molecules: Design, synthesis and preliminary SAR for antiplasmodial activity and falcipain-2 inhibition. Bioorg. Med. Chem. Lett. 2009;19:3229–32.

Fernández-de-la-Pradilla A, Royo S, Schirmeister T, Barthels F, Świderek K, González FV, et al. Impact of the Warhead of Dipeptidyl Keto Michael Acceptors on the Inhibition Mechanism of Cysteine Protease Cathepsin L. ACS Catal. 2023;13:13354–68.

Fuchs N, Meta M, Lantzberg B, Bros M, Ling Kuan S, Weil T, et al. Subnanomolar Cathepsin S Inhibitors with High Selectivity: Optimizing Covalent Reversible α‐Fluorovinylsulfones and α‐Sulfonates as Potential Immunomodulators in Cancer. ChemMedChem. 2023;18:e202300160.

Jung S, Fuchs N, Johe P, Wagner A, Diehl E, Yuliani T, et al. Fluorovinylsulfones and -Sulfonates as Potent Covalent Reversible Inhibitors of the Trypanosomal Cysteine Protease Rhodesain: Structure–Activity Relationship, Inhibition Mechanism, Metabolism, and In Vivo Studies. J. Med. Chem. 2021;64:12322–58.

Ren X-F, Li H-W, Fang X, Wu Y, Wang L, Zou S. Highly selective azadipeptide nitrile inhibitors for cathepsin K: design, synthesis and activity assays. Org. Biomol. Chem. 2013;11:1143.

Roush WR, Gwaltney SL, Cheng J, Scheidt KA, McKerrow JH, Hansell E. Vinyl Sulfonate Esters and Vinyl Sulfonamides: Potent, Irreversible Inhibitors of Cysteine Proteases. J. Am. Chem. Soc. 1998;120:10994–5.
